# HESREN: A Derivative-Informed Reservoir Framework for Detecting Transient Neural Events and Windowless Estimation of Dynamic Functional Connectivity

**DOI:** 10.1007/s12021-026-09792-3

**Published:** 2026-06-10

**Authors:** Ugur Kadak

**Affiliations:** 1 Gazi University, Ankara, Turkey; 2 Institute of Systems Neuroscience, Heinrich Heine University Düsseldorf, Düsseldorf, Germany

**Keywords:** Dynamic functional connectivity, Reservoir computing, Hermite-type neural operators, Transient event detection, Echo state networks, fMRI time series analysis

## Abstract

Dynamic functional connectivity (dFC) analysis in functional magnetic resonance imaging (fMRI) faces a fundamental challenge: conventional sliding-window methods must trade temporal resolution against statistical reliability, while rare transient neural events risk becoming undetectable when included in training data. We introduce HESREN (Hermite-Enhanced Software Reservoir Network), a novel framework integrating echo state networks with derivative-informed Hermite-type neural operators to enable windowless dFC estimation and leakage-free transient detection. HESREN employs a leaky-integrator reservoir that projects multivariate fMRI time series into high-dimensional state spaces, augmented with Gaussian-smoothed temporal derivatives to form enhanced feature vectors encoding value, velocity, and acceleration. Strict temporal partitioning trains all components exclusively on baseline segments while evaluating on complete time series, preserving transient events as out-of-distribution signals. Teacher-student distillation transfers the temporal precision of micro-window connectivity estimates into stable windowless operators via ridge-regularised linear readout; all hyperparameters and initialisation procedures are fully specified to ensure reproducibility. Validation on the NEBULA101 resting-state fMRI dataset across $N=50$ participants demonstrates consistent and substantial improvements over conventional methods. Transient event detection achieves AUC$=0.881\pm 0.025$ and average precision AP$=0.938\pm 0.018$, compared to AUC$=0.677\pm 0.074$ for raw-derivative baselines (Wilcoxon $W=1275$, $p<0.0001$, Cohen’s $d=2.84$), with phase-randomised surrogate testing confirming statistical robustness in all participants ($p=0.005$, $n=200$ surrogates). Comparison against mainstream dFC alternatives shows that HESREN statistically significant performance gains Gaussian Hidden Markov Models (AUC$=0.805\pm 0.088$), temporal convolutional networks (AUC$=0.815\pm 0.062$), LSTM autoregressive predictors (AUC$=0.580\pm 0.080$), and conventional sliding-window correlation (AUC$=0.690\pm 0.061$), with all advantages statistically significant ($p\le 0.028$). Windowless dFC trajectories attain lag-corrected correlation $r_{\textrm{lag}}=0.256\pm 0.019$ with micro-window teachers while providing 3–$5\times$ finer temporal resolution than 25-TR sliding windows. Network-level analysis reveals that HESREN detects transient events an average of 4.5 TR (9 s) earlier than sliding-window methods, selectively amplifies within-language-network coupling by $97\%$ and default-mode-network coupling by $119\%$ during detected events, and is the only evaluated method to yield a positive network segregation index ($\textrm{SI}=+0.038$), consistent with the known modular organisation of resting-state brain networks. HESREN overcomes fundamental limitations of sliding-window dFC through derivative-aware reservoir dynamics, offering a computationally efficient, mathematically principled framework for capturing transient neural reconfigurations with temporal precision previously improved in fMRI connectivity analysis. The modular architecture facilitates adaptation to diverse neuroimaging applications, from basic neuroscience to real-time clinical monitoring systems.

## Introduction

The human brain operates as a complex dynamical system characterized by continuous reconfiguration of functional networks across multiple temporal scales (van den Heuvel & Sporns, 2013; Deco et al., 2011). Understanding these dynamic patterns has emerged as a central challenge in modern neuroscience, particularly in the context of individual differences in cognitive abilities such as language aptitude (van den Heuvel & Hulshoff Pol, 2010; Finn et al., 2015). Resting-state functional magnetic resonance imaging (rs-fMRI) has revealed that spontaneous brain activity is organized into coherent networks of correlated regions, termed functional connectivity (FC) networks. FC is typically defined as the statistical dependence (e.g. correlation) between BOLD signals from different regions over time. Early studies treated FC as a stationary property of a scan, averaging correlations over the entire time series (Friston, 1994; Biswal et al., 1995). This static FC approach successfully identified canonical resting-state networks and individual traits (e.g. consistent default-mode and sensory networks, or subject-specific connectivity “fingerprints”). However, accumulating evidence suggests that connectivity patterns fluctuate over time, even in the resting brain (Hutchison et al., 2013; Allen et al., 2014; Calhoun et al., 2014; Lurie et al., 2020). In other words, FC is dynamic (varying on the scale of seconds to minutes), reflecting shifts in cognitive or physiological states – a concept sometimes termed the ”chronnectome”. Dynamic functional connectivity (dFC) analyses aim to characterize these time-varying connectivity states and have rapidly become a research frontier in neuroscience (Preti et al., 2017). Notably, dFC is hypothesized to provide information beyond static FC, potentially improving links to cognition and behavior (Fan et al., 2020; Luppi et al., 2019). These findings underscore that capturing connectivity dynamics is crucial for understanding brain function in health and disease.

The most common approach to estimate dFC is the sliding-window correlation (Sakoğlu et al., 2010). In this method, Pearson correlation (or another connectivity metric) is repeatedly computed within a short moving window across the fMRI time series, yielding a sequence of time-resolved connectivity matrices. Sliding-window analysis is simple and intuitive, but it suffers from important limitations. First, the choice of window length is inherently a trade-off: longer windows improve statistical reliability but cannot resolve brief connectivity changes, whereas shorter windows offer higher temporal resolution at the cost of noisy, unstable estimates (Leonardi & Van De Ville, 2015; Hindriks et al., 2016). The arbitrariness of the window size and shape can lead to inconsistent results – different studies often use windows ranging from  30 s to 2 min, affecting which dFC patterns are detected. Second, windowed correlations impose an implicit smoothing that can miss fast transients: if a rapid network reconfiguration occurs within a window, it gets averaged with surrounding time points and thus diluted. Conversely, if windows are too short, correlation estimates can fluctuate wildly due to sampling variability, producing spurious patterns not driven by neural activity (Lindquist et al., 2014). Third, standard sliding-window analysis assumes a single connectivity state per window and may not detect asynchronous or overlapping changes across different subnetworks. These issues have prompted concerns about the reliability and interpretation of sliding-window dFC. Empirically, test–retest studies report that many dFC metrics have modest reliability (Noble et al., 2019), and it remains debated to what extent observed dFC reflects true neural dynamics versus noise or preprocessing artifacts. In summary, static FC analysis ignores meaningful temporal variations, yet naive sliding-window dFC approaches face biases and sensitivity limits, motivating the search for more principled methods.

To overcome the limitations of sliding windows, a variety of alternative techniques for dFC have been developed. Some methods forego windows altogether by focusing on events in the BOLD time series. For example, point-process analysis detects moments of suprathreshold BOLD peaks and evaluates connectivity from the co-occurrence of such events (Tagliazucchi et al., 2012). This data-driven approach, introduced by Tagliazucchi et al. (2012), effectively binarizes the time series and has been shown to recover resting-state networks similar to traditional FC. Similarly, co-activation pattern (CAP) analysis identifies brief instances when a specific set of regions jointly activate, revealing recurring connectivity patterns on sub-second timescales (Liu & Duyn, 2013). These event-based approaches circumvent the need to predefine a window length, capturing fast transients that sliding windows might miss. Other approaches explicitly model state transitions in connectivity. Hidden Markov Models (HMMs), for instance, assume the fMRI time series alternates among a set of discrete latent connectivity states, with each state characterized by a unique covariance structure. Vidaurre et al. (Vidaurre et al., 2017) demonstrated that HMMs can effectively discover recurrent brain states from rs-fMRI, revealing a rich temporal hierarchy of connectivity patterns underlying the “dynamic connectome.” One such technique detected abrupt FC changes by modeling correlation matrices as piecewise constant segments (Cribben et al., 2012). More recently, state-space models and Kalman filter approaches have been used to continuously track time-varying connectivity as a smoothly evolving parameter, balancing flexibility with statistical regularization (Kabbara et al., 2020). Beyond these algorithmic advances, researchers have also reconsidered how dFC is represented. For example, an “edge-centric” perspective was proposed by Faskowitz et al. (Faskowitz et al., 2020), in which each pairwise connection’s time series (the z-scored edge weight at each time point) is analyzed; this approach retains maximal temporal information by treating the dynamic connectivity itself as the signal of interest rather than summarizing it into coarse states. Overall, these diverse methods – spanning event detection, pattern clustering, switching-state models, and novel representations – have enriched the dFC field. They report that brain connectivity continually reconfigures into motifs associated with specific conditions and stimuli. Crucially, dFC analysis has revealed links between connectivity dynamics and various physiological and clinical variables. For instance, distinct dFC patterns have been associated with cognitive flexibility (Douw et al., 2016), emotional affect (Tobia et al., 2014), and developmental or aging processes (Qin et al., 2015). Abnormal dFC temporal properties have been reported in neurological and psychiatric conditions including schizophrenia (Damaraju et al., 2014), Alzheimer’s disease (Gu et al., 2020), autism (Zhu et al., 2017), and major depression (Demirtaş et al., 2016), suggesting that chronnectomic biomarkers hold clinical relevance. Despite this progress, many dFC methods still face trade-offs between temporal precision and statistical robustness, and there remains a need for frameworks that can capture brief, transient connectivity events without sacrificing interpretability or requiring extensive parameter tuning.

### Neural Networks and Reservoir Computing Foundation for dFC

Recently, data-driven machine learning approaches have been explored to model dynamic connectivity more flexibly. In particular, researchers have begun applying deep neural networks to recognize and predict dFC patterns from fMRI time series. For example, Fan et al. (Fan et al., 2020) developed an end-to-end deep model combining convolutional and recurrent (LSTM) layers to learn spatiotemporal features of dFC directly, outperforming conventional feature-based methods in classifying sex and predicting intelligence from rs-fMRI. Such results demonstrate the promise of deep learning to exploit the rich information in dFC sequences that simpler statistical measures might overlook. However, most deep learning applications to date treat dFC analysis as a supervised learning task (e.g. classification or regression using dFC as input or output), rather than attempting to estimate dynamic connectivity itself in an unsupervised manner. A different but related machine-learning trend with great potential for dFC is the rise of neural operator learning. Traditional neural networks approximate mappings between finite-dimensional vectors, but neural operators aim to learn mappings between functions (infinite-dimensional function spaces) (Chen & Chen, 1995). The theoretical foundations date back to the universal approximation theorems for operators, which showed that a neural network with appropriate architecture can approximate any nonlinear operator to arbitrary accuracy (given sufficient resources), extending the classic universal approximation property from functions to functionals (Chen & Chen, 1995). This concept has recently been popularized by frameworks like DeepONet (Lu et al., 2021) and the Fourier Neural Operator (Li et al., 2020), which have achieved success in learning the solution operators of partial differential equations and other complex systems. In essence, these models take functions (e.g. initial or boundary conditions, temporal signals) as inputs and output an entire function (e.g. a solution field or a time-varying response), using architectures that blend neural networks with integral kernels or basis function expansions. Operator-learning approaches are attractive for dynamic connectivity because they can, in principle, learn a mapping from the fMRI time series to a time-resolved connectivity trajectory in one step – i.e. treating dFC estimation itself as a regression problem on function space, rather than a sliding correlation problem. Furthermore, emerging research on derivative-informed models provides additional inspiration: incorporating known derivatives or physical constraints into network training has been shown to improve learning efficiency and accuracy. For example, physics-informed neural networks (PINNs) embed differential equation residuals into the loss function so that the learned function obeys physical laws (Raissi et al., 2019). O’Leary-Roseberry et al. (O’Leary-Roseberry et al., 2024) recently introduced a Derivative-Informed Neural Operator (DINO) that trains a network to match not only the target operator’s outputs but also its Jacobian (sensitivity) with respect to inputs, thereby enforcing correctness of learned derivatives. In their experiments, injecting derivative information dramatically improved the operator’s accuracy and data efficiency, especially under limited training data. These advances in neural operators and derivative-informed learning have yet to be fully explored in neuroimaging, but they point toward a new frontier for dFC analysis: network models that directly learn the functional mapping from BOLD time series to dynamic connectivity, enriched by derivative features.

Another pillar of our approach is reservoir computing, a powerful RNN paradigm for sequence modeling. Echo State Networks (ESNs) (Jaeger, 2001) and Liquid State Machines (LSMs) (Maass et al., 2002) were both introduced in the early 2000s as methods to harness high-dimensional recurrent dynamics without backpropagating through time. In an ESN, a large recurrent network (the “reservoir”) with fixed random weights serves as a non-linear excitable medium, and only the output weights are trained (typically by linear regression) to map the reservoir’s state trajectory to the desired output signal. This yields a fast and stable training process while preserving rich dynamics in the reservoir (Lukoševičius & Jaeger, 2009). Subsequent work has extended reservoir computing in various ways: Deep Reservoir Computing stacks multiple reservoirs in layers to progressively extract higher-level temporal features, which Gallicchio et al. (Gallicchio et al., 2017) found can improve performance on complex tasks. Physical reservoir computing leverages analog systems (e.g. optical, mechanical, or biophysical systems) as reservoirs (Tanaka et al., 2019), exploiting natural dynamics for computation and achieving remarkable speed or energy efficiency in some cases. These developments show the versatility of reservoir computing in capturing temporal patterns. In the context of fMRI, reservoir networks offer an appealing way to model brain dynamics because they can learn temporal relationships without explicit sliding windows. The reservoir’s state *h*(*t*) can be viewed as a nonlinear embedding of the history of the BOLD signals up to time *t*, which a readout can use to estimate time-dependent connectivity. Notably, reservoirs can naturally integrate information over varying timescales via their recurrent feedback loops, potentially capturing both slow drifts and fast transients in a unified model. Preliminary studies have applied RNNs and ESNs to neuroimaging data (e.g. for prediction of future brain states or classification of cognitive conditions), suggesting that these models can capture subtle temporal structure in BOLD signals. However, standard RNNs or ESNs have not yet been widely used to compute connectivity measures themselves. This is the opportunity we aim to seize by marrying reservoir computing with neural operator learning.

### Research Gap and Main Contributions

Based on this literature review, we identify a clear gap that motivates our work. On one hand, traditional dFC methods (sliding windows and even many advanced variants) struggle to detect short-lived, high-frequency connectivity changes without sacrificing confidence in the estimates. They either rely on user-defined windows or specific assumptions (hidden states, change points, etc.) that can limit their sensitivity or interpretability. On the other hand, modern machine learning approaches (deep networks and neural operators) offer a means to learn complex mappings and leverage derivative information, but these techniques have not yet been fully applied to capturing functional connectivity dynamics in fMRI. In particular, no existing framework combines the adaptive sequence processing of reservoir RNNs with an operator-learning view of connectivity estimation. We lack a windowless, learnable model that can ingest fMRI time series and output time-resolved connectivity on the fly, with the ability to detect rapid connectivity fluctuations as well as gradual trends. Furthermore, prior dFC studies have largely not incorporated temporal derivative features of the signals, despite evidence that such features could enhance detection of state transitions. The author’s recent works pioneered the idea of derivative-informed neural operators in imaging (Kadak, 2022, 2025, 2026a; Coroianu et al., 2022; Kadak et al., 2023), but these remain theoretical or methodological contributions that have yet to be integrated into a full pipeline for fMRI connectivity analysis.

In this paper, we address the above gap by introducing the Hermite-type Software REServoir Network (HESREN) for dynamic fMRI connectivity analysis. HESREN is a novel framework that unites reservoir computing with neural operator learning and derivative-awareness to provide windowless, high-fidelity dFC estimates. In building HESREN, we incorporate several innovations and contributions:

1. **Derivative-informed reservoir framework:** We propose a new readout architecture for the ESN inspired by the Hermite Neural Network operator (HNN) introduced by the author (Kadak, 2026b). Rather than using a simple linear regression on reservoir states, HESREN’s readout employs a Hermite-type operator that takes as input both the reservoir states and their time derivatives. By matching both the values and the temporal derivatives of the reservoir state trajectory, this Hermite operator achieves a higher-order approximation to the target dynamic connectivity function. Intuitively, this means our model can capture not only the level of connectivity changes but also the velocity and acceleration of those changes, improving sensitivity to sharp transitions.
2. **Windowless Dynamic Connectivity via Reservoir Computing:** HESREN provides continuous dynamic connectivity estimates without relying on any fixed-size sliding window. The ESN reservoir, with its recurrent architecture, serves as a “memory” of the recent BOLD history, and our learned readout function maps the current reservoir state (and its derivatives) directly to an instantaneous connectivity matrix. This approach can be seen as learning a neural operator *F* such that $C(t) = F[x(1:t)]$, mapping the time series up to time *t* to the current connectivity *C*(*t*). By learning this mapping from data (through a teacher–student training protocol described in Section “HESREN — A Derivative-Informed Reservoir–Operator Framework”), HESREN adapts to the intrinsic timescales of the fMRI signals. Short-lived but reproducible co-fluctuation events can be captured as fast changes in the output without averaging them out.
3. **Leakage-Free Training for Event Detection:** A key contribution is a rigorous training protocol that prevents information leakage from transient events into the model during training. If the model is trained on data containing event-related spikes, it could inadvertently learn these specific events rather than the general dynamics, leading to overfitting and diminished ability to detect novel events. We address this by baseline-event segmentation: all high-amplitude transient events in the training data are identified via outlier detection on the BOLD time series, and those time points are excluded from readout training. This protocol is validated by phase-randomised surrogate testing ($n=200$ surrogates, $p=0.005\pm 0.000$ across $N=50$ participants).
4. **Graph-theoretic network characterization:** Comprehensive topology analysis reveals that HESREN enhances hub-periphery organisation: degree centrality of dominant hubs increases by 25–$30\%$ relative to teacher networks, with eigenvector centrality exhibiting stronger prestige differentiation (Pearson $r(\textrm{EC},\textrm{DC})=0.91$ versus 0.78 for teacher, 0.72 for sliding-window). Under common absolute *z*-thresholding, HESREN produces denser graphs ($D=0.621$ versus $D=0.470$ for teacher, $+32\%$ increase) with more integrated topology: shorter paths, higher transitivity, and greater global efficiency (Figs. 11-12). Graph-spectral analysis (Fig. 13) confirms small-world organisation ($\sigma =\gamma /\lambda =1.91>1$) with positive degree assortativity ($r\approx 0.40$) and non-trivial algebraic connectivity ($\lambda _2=0.188$).
5. **Integration of Multi-Scale Dynamics:** The HESREN reservoir and Hermite operator together allow modelling of multi-scale connectivity changes. The reservoir’s internal memory (controlled by leak rate and spectral radius hyperparameters) can span several dozen seconds, capturing slow fluctuations (e.g. trends related to vigilance or physiology), while the inclusion of derivative features targets the rapid, fleeting changes. Gaussian smoothing of reservoir states before differentiation implements a tunable short-term integration that improves numerical stability of derivatives.
6. **Multi-subject validation and competitive benchmarking.** The framework is validated on an extended cohort of $N=50$ participants from the NEBULA101 dataset, demonstrating consistent performance (AUC$=0.881\pm 0.025$, AP$=0.938\pm 0.018$, $p_{\textrm{null}}=0.005\pm 0.000$ in all participants). A direct comparison against mainstream dFC methods on all participants shows that HESREN statistically significant performance gains Gaussian Hidden Markov Models (AUC$=0.805\pm 0.088$), temporal convolutional networks (AUC$=0.815\pm 0.062$), LSTM autoregressive predictors (AUC$=0.580\pm 0.080$), and conventional sliding-window correlation (AUC$=0.690\pm 0.061$), with all advantages confirmed by Wilcoxon signed-rank tests ($p\le 0.028$, Cohen’s *d* up to 3.89).
7. **Neuroscientific network interpretation.** Network-level analysis maps the 12 ROIs to four canonical functional systems (language, executive control, default mode, and auditory cortex) and demonstrates that HESREN’s derivative-informed processing yields biologically interpretable connectivity estimates. HESREN detects transient events an average of 4.5 TR (9 s) earlier than sliding-window methods, selectively amplifies within-language-network coupling by $97\%$ and default-mode-network coupling by $119\%$ during detected events, and is the only evaluated method to yield a positive network segregation index ($\textrm{SI}=+0.038$), consistent with the known modular organisation of resting-state brain networks.

In summary, HESREN is a windowless, derivative-informed neural network framework for dynamic connectivity that brings together the complementary strengths of reservoir computing (efficient sequence modelling), Hermite-type neural operators (higher-order function approximation using derivatives), and task-specific training protocols (teacher-student distillation and leakage prevention). To our knowledge, this is the first approach to train a neural network to directly output dynamic correlation estimates from fMRI data in an online fashion, validate this framework across a cohort of $N=50$ participants, and benchmark it against mainstream deep temporal and probabilistic dFC methods. The contributions of this work include: (1) formulating a Hermite-type ESN readout that improves dynamic connectivity estimation by incorporating temporal derivatives of hidden states; (2) demonstrating windowless dFC estimation that outperforms conventional sliding-window correlations in temporal fidelity and event sensitivity; (3) introducing a leakage-free training procedure that enables reliable detection of novel connectivity events; (4) empirically validating that HESREN statistically significant performance gains Hidden Markov Models, LSTM, and TCN baselines ($p\le 0.028$); (5) providing full algorithmic specification and hyperparameter documentation to ensure reproducibility; and (6) demonstrating that HESREN recovers biologically meaningful network organisation, including a 9-second temporal detection advantage and positive network segregation index, consistent with known resting-state network structure.

The rest of this paper is organized as follows. Section “Datapreprocessing” describes the fMRI dataset and preprocessing steps used in this study, including region-of-interest definition and baseline segment identification. Section “HESREN — A Derivative-Informed Reservoir–Operator Framework” details the HESREN framework: we present the ESN architecture and notation, define the Hermite-type Neural Network Operator and its discrete implementation, and outline the training procedure (teacher–student distillation and leakage prevention) that yields the dynamic connectivity operator, and complete algorithmic specification with hyperparameter documentation. Section “Results: Methodological Validation and Multi-Subject Analysis” reports experimental results, comparing HESREN’s dynamic connectivity estimates to sliding-window and short-window baselines on both qualitative and quantitative metrics; extended multi-subject validation across $N=50$ participants; comparison against HMM, LSTM, TCN, and SWC methods; advanced network-level analysis with neuroscientific interpretation; and graph-theoretical characterisation of HESREN connectivity patterns. Section “Discussion and Future Directions” provides a discussion of the implications of our findings, methodological innovations in context, current limitations, and avenues for future research. Section “Information Sharing Statement” concludes the paper.

## Datapreprocessing

The NEBULA101 (Neuro-behavioural Understanding of Language Aptitude) dataset (Rampinini et al., 2025) is an open resource comprising behavioral measures and multi modal brain imaging data (including resting-state fMRI) from 101 healthy adults. This dataset specifically targets individual differences in language aptitude the capacity to learn additional languages by providing rich cognitive and neural phenotyping in a diverse sample. We focus here on the resting-state fMRI component to model dynamic functional connectivity (dFC), i.e. the temporal fluctuations in inter-regional brain coupling. The rationale for using fMRI-based dFC is that static connectivity averages may obscure meaningful variations linked to cognitive traits. Language aptitude is thought to emerge from complex, dynamic interactions among brain regions supporting language and domain-general processes. By examining time-resolved connectivity patterns, we can capture transient network configurations that might correlate with aptitude, potentially revealing individual fingerprints of brain network flexibility.

All fMRI data underwent standard preprocessing using the FSL (FMRIB Software Library) toolbox. The pipeline included motion correction (MCFLIRT), slice timing correction, spatial smoothing witha 6 mm FWHM Gaussian kernel, high-pass temporal ltering (cuto = 100 s), and brain extraction (BET). Functional images were then registered to MNI152 standard space using FLIRT and FNIRT for linear and non-linear alignment, respectively. Importantly, we also applied ICA-based artifact removal (FIX) to minimize physiological and scanner-related noise. For each participant, a binary brain mask was generated for time series extraction. These steps ensure that the resulting data are temporally and spatially standardized, artifact-reduced, and aligned across subjects, facilitating reliable computation of dynamic functional connectivity metrics across the cohort.

NEBULA101 resting-state fMRI data were acquired on a 3T Siemens Prisma scanner using a multiband echo-planar imaging (EPI) sequence with the following parameters:

- Repetition time (TR): 2.0 seconds
- Echo time (TE): 30 ms
- Flip angle: 90
- Voxel size: $2 \times 2 \times 2$ ${mm}^{3}$ isotropic
- Matrix size: $$96 \times 96$$
- Number of slices: 60 (axial acquisition)
- Multiband acceleration factor: 3
- Scan duration: Approximately 10 minutes (300 volumes).

The 2-second TR provides adequate temporal sampling for hemodynamic responses (typical duration 4-8 seconds) while enabling computation of temporal derivatives with reasonable signal-to-noise ratio. The 10-minute acquisition duration yields sufficient data ($T = 300$ timepoints) for reliable estimation of HESREN parameters while remaining within tolerable limits for subject compliance and motion.

### Region of Interest (ROI) Definition

Spatial parcellation represents a critical design choice in functional connectivity analysis, balancing the competing demands of spatial coverage, anatomical specificity, and statistical power. We employed a data-driven ROI selection strategy tailored to individual subjects, complemented by anatomically-defined backup coordinates when data-driven selection proved suboptimal. For each subject, we computed the temporal standard deviation map—the voxel-wise standard deviation of the preprocessed BOLD signal across time:

$$\begin{aligned} \sigma _v = \sqrt{\frac{1}{T-1} \sum _{t=1}^T \big (x_v(t) - \bar{x}_v\big )^2}, \end{aligned}$$

where $x_v(t)$ is the BOLD signal at voxel *v* and time *t*, and $\bar{x}_v$ is the temporal mean. Regions with high temporal variability typically correspond to gray matter areas exhibiting strong functional fluctuations, while low-variability regions often reflect white matter, CSF, or areas with poor signal quality. We identified local maxima (peaks) in the temporal standard deviation map as candidate ROI centers, subject to the following constraints: (i) Only voxels within the subject-specific brain mask were considered; (ii) ROI centers were required to be separated by at least $d_{\min } = 2 \times r = 16$ mm in Euclidean distance, where $r = 8$ mm is the sphere radius (described below). This prevents substantial spatial overlap between spherical ROIs; (iii) If fewer than the target number of ROIs ($N_{\text {ROI}} = 12$) could be identified with the initial separation constraint, $d_{\min }$ was iteratively reduced by 25% (multiplied by 0.75) up to 3 times until sufficient ROIs were obtained. When data-driven selection failed to identify sufficient ROIs (e.g., due to excessive motion artifacts or poor image quality), we employed a predefined set of anatomically-motivated coordinates based on meta-analytic evidence for language-relevant networks and domain-general control systems. These coordinates were defined in MNI152 space and included:

#### Language Network (Left Hemisphere)

- Inferior frontal gyrus, pars opercularis (Broca’s area / BA44): (-48, 12, 14)
- Posterior superior temporal gyrus (Wernicke’s area): (-60, -42, 12)
- Middle temporal gyrus: (-60, -30, -6)
- Angular gyrus: (-45, -66, 30)

#### Executive Control and Salience Networks

- Dorsolateral prefrontal cortex (left DLPFC): (-44, 36, 30)
- Anterior insula (left aINS): (-32, 22, 2)
- Dorsal anterior cingulate cortex (dACC): (0, 24, 36)

#### Default Mode Network

- Medial prefrontal cortex (mPFC): (0, 52, -2)
- Posterior cingulate cortex (PCC): (0, -52, 26)
- Angular gyrus (DMN component): (-46, -66, 28)

#### Auditory Cortex (Bilateral)

- Left Heschl’s gyrus: (-42, -22, 8)
- Right Heschl’s gyrus: (42, -22, 8)

These coordinates are selected from established atlases and meta-analyses (Glasser et al., 2016) to sample key nodes of networks relevant to language processing and domain-general cognition. The fallback mechanism ensures that all subjects have consistent ROI coverage even when data quality precludes fully data-driven selection. Time series were extracted from spherical regions (radius $r = 8$ mm) centered at each selected coordinate using Nilearn’s NiftiSpheresMasker. For each ROI, the mean signal across all voxels within the sphere is computed at each time point. The resulting data matrix for each subject was $\textbf{X} \in \mathbb {R}^{T \times R}$, where $T \approx 300$ timepoints and $R = 12$ ROIs. These standardized time series served as input to the HESREN pipeline.

## HESREN — A Derivative-Informed Reservoir–Operator Framework

### Overview and Notation

The Hermite-Enhanced Software Reservoir Network (HESREN) is designed to uncover transient neural events and dynamic connectivity patterns in fMRI data without relying on sliding windows. Traditional sliding-window approaches suffer from an inherent trade-off between temporal resolution and statistical reliability: narrow windows provide fine temporal precision but at the cost of noisy connectivity estimates, while wider windows yield stable estimates but obscure rapid neural reconfigurations that may be behaviorally relevant (Hutchison et al., 2013; Preti et al., 2017). This fundamental constraint has limited our ability to capture the fast, transient network reorganizations that characterize real brain dynamics during rest and task performance. Conceptually, HESREN integrates two powerful components: (i) a *leaky-integrator Echo State Network* (ESN) that projects multivariate fMRI time series into a high-dimensional dynamical state space, and (ii) a *derivative-informed trainable neural operator* that serves as a flexible readout to capture rapid temporal modulations. Rather than computing connectivity estimates from fixed temporal windows, our framework learns to extract dynamic connectivity patterns directly from high-dimensional reservoir states and their temporal derivatives. This paradigm shift enables windowless estimation of time-varying connectivity while preserving sensitivity to brief neural events that would be smoothed away by conventional methods.

The theoretical foundation of HESREN rests on three key insights from dynamical systems theory and functional analysis. First, ESNs provide a natural framework for capturing the temporal dependencies inherent in fMRI time series through their fading memory property (Jaeger, 2001). The reservoir’s recurrent dynamics implicitly integrate past information, creating a rich representation of temporal context without requiring gradient-based training of recurrent weights. Second, incorporating temporal derivatives—inspired by Hermite type sampling theory (Corso, 2025; Kadak, 2026c)—allows the readout mechanism to be sensitive not only to instantaneous states but also to rates of change and acceleration, which are crucial signatures of transient events. Third, the teacher-student distillation paradigm enables us to transfer the temporal precision of micro-window connectivity estimates into a continuous, windowless operator that maintains both accuracy and temporal resolution.

### Reservoir Computing Foundation: Echo State Network Architecture

The reservoir computing paradigm, pioneered by Jaeger and Haas (Jaeger, 2001) and independently by Maass et al. (Maass et al., 2002), offers a computationally efficient alternative to fully trainable recurrent neural networks. In reservoir computing, a high-dimensional dynamical system with fixed, randomly initialized recurrent connections serves as a rich temporal feature extractor. Only the output layer (readout) is trained, typically through simple linear regression, which dramatically reduces computational cost while maintaining expressive power for temporal sequence processing. HESREN employs a leaky-integrator ESN with elementwise $\tanh$ nonlinearity,

$$\begin{aligned} h_t = (1-\alpha )h_{t-1} + \alpha \,\tanh \!\big (W_{\!\textrm{in}}\tilde{x}_t + W h_{t-1} + b\big ), \end{aligned}$$

where:

- ${h}_t \in \mathbb {R}^N$ is the reservoir state vector at time *t*
- ${W}_{in} \in \mathbb {R}^{N \times p}$ is the input weight matrix (fixed, randomly initialized)
- ${W} \in \mathbb {R}^{N \times N}$ is the recurrent reservoir matrix (fixed, sparse)
- ${b} \in \mathbb {R}^N$ is the bias vector
- $\alpha \in (0,1]$ is the leak rate controlling temporal integration.

The leak rate $\alpha$ plays a crucial role in determining the reservoir’s temporal memory profile. Smaller values of $\alpha$ result in longer effective memory by allowing past states to decay more slowly, while larger values produce faster adaptation to new inputs. For fMRI applications with typical repetition times (TR) of 1-2 seconds, we empirically find that moderate leak rates in the range $\alpha \in [0.25, 0.6]$ provide an optimal balance between responsiveness to new inputs and retention of temporal context. This range corresponds to effective memory horizons of approximately 3-8 TRs, which aligns well with the temporal scales of hemodynamic responses and short-term neural state fluctuations. The element-wise $\tanh$ nonlinearity ensures that reservoir activations remain bounded, preventing numerical instabilities during long sequences. The hyperbolic tangent function also provides a smooth, differentiable saturation that enhances the reservoir’s ability to capture complex nonlinear relationships in the input data. The bias term *b* is typically initialized to zero or drawn from a small normal distribution, providing additional representational flexibility without introducing substantial variance.

#### Reservoir Initialization and the Echo State Property

The echo state property requires that the reservoir’s dynamics asymptotically forget initial conditions and depend primarily on recent input history. Mathematically, this property is guaranteed when the spectral radius $\rho (W)$ (the largest absolute eigenvalue of *W*) satisfies certain boundedness conditions relative to the input scaling and nonlinearity. We initialize the reservoir matrices according to the following protocol. The input weight matrix $W_{\text {in}}$ is drawn from a Gaussian distribution:

$$\begin{aligned} W_{\text {in}}^{(i,j)} \sim \mathcal {N}\big (0, \sigma _{\text {in}}^2\big ), \quad \text {where} \quad \sigma _{\text {in}} = \frac{\gamma _{\text {in}}}{\sqrt{p}}, \end{aligned}$$

with input scaling parameter $\gamma _{\text {in}} \in [0.1, 2.0]$ controlling the strength of external drive. The normalization by $\sqrt{p}$ ensures that the total input contribution remains stable as the number of ROIs increases, following standard practices in neural network initialization. The recurrent matrix *W* is initialized as a sparse random matrix:

$$\begin{aligned} W^{(i,j)} \sim {\left\{ \begin{array}{ll} \mathcal {N}(0, \sigma _w^2) & \text {with probability } \rho _{\text {sparse}} \\ 0 & \text {otherwise,} \end{array}\right. } \end{aligned}$$

where $\rho _{\text {sparse}} \in [0.05, 0.15]$ determines the connectivity density. Sparse connectivity serves multiple purposes: it reduces computational cost (particularly for large reservoirs), introduces structural heterogeneity that can enhance reservoir expressivity, and helps avoid pathological attractor states that can arise in fully connected networks. After initialization, the reservoir matrix is rescaled to achieve a desired spectral radius:

$$\begin{aligned} W\leftarrow \frac{\gamma _w}{\rho (W)} W, \end{aligned}$$

where $\gamma _w \in [0.5, 1.2]$ is the target spectral radius and $\rho (W)$ denotes the spectral radius before rescaling. The spectral radius is computed using power iteration or Arnoldi methods. Setting $\gamma _w$ slightly below unity ($\gamma _w \approx 0.9$–0.95) typically ensures the echo state property while maintaining rich dynamics. Values closer to unity enhance memory capacity and temporal expressivity, while smaller values prioritize stability and reduce sensitivity to initialization. The interplay between $\alpha$, $\gamma _w$, and $\gamma _{\text {in}}$ defines the reservoir’s effective operating regime. High input scaling with moderate spectral radius yields input-driven dynamics, where the reservoir closely tracks input variations. Conversely, high spectral radius with moderate input scaling promotes autonomous dynamics, where recurrent interactions play a dominant role. For fMRI dFC estimation, we favor a balanced regime that allows both input-driven responses (capturing immediate signal changes) and recurrent integration (smoothing noise and maintaining temporal context).

#### Computational Efficiency and Scalability

A key advantage of reservoir computing is computational efficiency. The forward pass through the reservoir involves a single sparse matrix-vector multiplication per time step, with computational complexity $\mathcal {O}(T N_{\text {nz}})$, where *T* is the sequence length and $N_{\text {nz}} \ll N^2$ is the number of non-zero entries in the sparse matrix *W*. For typical fMRI sequences ($T \approx 200$–500 TRs) and reservoir sizes ($N \in [200, 1000]$) with 5–10% connectivity, state generation completes in seconds on standard hardware. Memory requirements scale linearly with reservoir size and sequence length, as we store the full trajectory $\{{h}_t\}_{t=1}^T$ for subsequent readout training. For a reservoir with $N=500$ nodes and a sequence of $T=300$ time points, the state matrix occupies approximately $500 \times 300 \times 8$ bytes $\approx$ 1.2 MB in double precision, which is negligible compared to typical fMRI data storage requirements. This favorable scaling enables HESREN to process multi-subject datasets efficiently even on modest computational resources.

### Hermite-Type Neural Network Operators

Following the construction introduced by Kadak (Kadak, 2026b), the Hermite-type neural network (HNN) operator extends classical Hermite interpolation to a kernel–normalized, neural feature setting. Instead of relying only on pointwise function values, the operator blends local Taylor information (function and its partial derivatives up to order *r*) around grid nodes and aggregates them through a positive density kernel with a partition-of-unity denominator. This yields a multivariate, derivative-aware approximant that remains stable near boundaries and admits $L^\infty$ (Hermite) and $L^p$ (Kantorovich) convergence under mild smoothness.

The Hermite-Type Neural Network Operator (HNN) represents the core innovation that distinguishes HESREN from standard reservoir computing approaches. While conventional ESN readouts employ simple linear regression from reservoir states to target outputs, HNNO augments this mapping by incorporating temporal derivative information. This design is inspired by classical Hermite interpolation in numerical analysis, where polynomial approximations match not only function values but also derivatives at interpolation nodes, resulting in higher-order accuracy. HESREN framework motivates a readout that uses reservoir states together with their temporal derivatives, allowing the model to capture both slow drifts and sharp reconfigurations in dynamic connectivity while preserving robustness via localized normalization and, when needed, Kantorovich-style averaging. We next detail the operator forms and their properties that underlie our implementation.

For a multivariate function $f: \mathbb {R}^s \rightarrow \mathbb {R}$ possessing *r*-times continuous partial derivatives (i.e., $f \in C_b^r(\mathbb {R}^s)$, the space of bounded functions with continuous partial derivatives up to order *r*), Kadak(Kadak, 2026b) introduced the Hermite-type Neural Network operator of order *r* as:

1 $$\begin{aligned} (H^{(r)}_{n}f)(\underline{x}) = \frac{\displaystyle \sum \limits _{\underline{k}} \left( \sum \limits _{|\underline{\alpha }| \le r} \frac{1}{j!} \left( D^{j}f\right) \left( \frac{\underline{k}}{n}\right) \left( \underline{x} - \frac{\underline{k}}{n}\right) ^{\underline{\alpha }} \right) \Psi _\sigma (n\underline{x}-\underline{k})}{\displaystyle \sum \limits _{\underline{k}} \Psi _\sigma (n\underline{x}-\underline{k})}, \quad \underline{x} \in \mathbb {R}^s, \end{aligned}$$

where:

- $\underline{x} = (x_1, \ldots , x_s) \in \mathbb {R}^s$ represents the evaluation point in *s*-dimensional space,
- $\underline{k} = (k_1, \ldots , k_s) \in \mathbb {Z}^s$ indexes a regular lattice of sampling points scaled by $n \in \mathbb {N}$,
- $D^{j}f = \frac{\partial ^{j}f}{\partial ^{\alpha _1}_{x_1}\partial ^{\alpha _2}_{x_2}\cdots \partial ^{\alpha _s}_{x_s}}$ denotes the *j*-th order partial derivative,
- $\underline{\alpha } = (\alpha _1, \ldots , \alpha _s)$ is a multi-index with $|\underline{\alpha }| = \sum _{i=1}^s \alpha _i \le r$, where $j = |\underline{\alpha }|$,
- $\Psi _\sigma :\mathbb {R}^s \rightarrow \mathbb {R}_+$ is a multivariate density function (kernel), typically chosen as a product of sigmoidal activations or Gaussian functions, providing localized weighting around lattice points.

HNN satisfies the approximation guarantee $\lim _{n \rightarrow \infty } \Vert H^{(r)}_{n}f - f \Vert _{\infty } = 0,$ where $\Vert \cdot \Vert _{\infty }$ denotes the supremum norm. This result guarantees that as the sampling density *n* increases, the HNN operator uniformly reconstructs the target function. The convergence rate depends on the smoothness of *f* and the order *r*: higher-order derivatives provide faster convergence for sufficiently smooth functions.

### HESREN Architecture

In the context of dynamic fMRI analysis, temporal derivatives carry crucial information about the nature and timing of neural state transitions. A gradual drift in connectivity patterns produces small first-order changes over many time points, while an abrupt reconfiguration—such as a transition between cognitive states or a physiological artifact—generates a sharp spike in the first derivative and potentially non-zero second derivatives. By explicitly incorporating these derivative features into the readout mechanism, HNN becomes sensitive to the temporal structure of state changes, not merely their magnitude. HESREN adapts the Hermite operator framework to reservoir computing through a discrete-time implementation suitable for fMRI time series analysis. The conceptual mapping is as follows: In the abstract operator framework (Eq. 1), the spatial domain $\underline{x} \in \mathbb {R}^s$ and lattice index $\underline{k} \in \mathbb {Z}^s$ are general multidimensional constructs. HESREN specializes to the univariate temporal case ($s=1$) with discrete time points $t \in \{1, \ldots , T\}$:

- The ”lattice points” *k*/*n* become discrete time indices *t*,
- The density function $\Psi _\sigma$ corresponds to temporal smoothing (Gaussian filtering),
- The integration in HKNN maps to discrete summation over local windows.

The reservoir state $\textbf{h}_t \in \mathbb {R}^N$ serves as the multivariate function *f* that the Hermite operator approximates. Rather than a single scalar time series, we have an *N*-dimensional trajectory:

$$\begin{aligned} f(\underline{x}) \quad \longrightarrow \quad \textbf{h}:\mathbb {R} \rightarrow \mathbb {R}^N, \quad t \mapsto \textbf{h}_t. \end{aligned}$$

Each reservoir dimension evolves in time, and HESREN’s readout must reconstruct target outputs (e.g., next-step predictions $\hat{\textbf{x}}_{t+1}$, connectivity estimates $\hat{C}_{ij}(t)$) from this high-dimensional trajectory. The continuous partial derivatives $D^j \textbf{f}$ in the abstract operator are implemented through discrete backward differences up to order *r*. For any order $j \in \{1, 2, \ldots , r\}$, the *j*-th order discrete derivative is recursively defined as:

$$\begin{aligned} D^1 \textbf{h}_t&\approx \Delta \textbf{h}_t = \textbf{h}_t - \textbf{h}_{t-1}, \\ D^j \textbf{h}_t&\approx \Delta ^j \textbf{h}_t = \Delta ^{j-1} \textbf{h}_t - \Delta ^{j-1} \textbf{h}_{t-1}, \quad j = 2, 3, \ldots , r. \end{aligned}$$

Expanding the recursion explicitly, we obtain the general binomial form:

$$\begin{aligned} \Delta ^j \textbf{h}_t = \sum _{\ell =0}^{j} (-1)^{j-\ell } \left( {\begin{array}{c}j\\ \ell \end{array}}\right) \textbf{h}_{t-\ell }, \quad j = 1, 2, \ldots , r, \end{aligned}$$

where $$\left( {\begin{array}{c}j\\ \ell \end{array}}\right) $$ denotes the binomial coefficient. For instance:

$$\begin{aligned} \Delta ^1 \textbf{h}_t&= \textbf{h}_t - \textbf{h}_{t-1}, \\ \Delta ^2 \textbf{h}_t&= \textbf{h}_t - 2\textbf{h}_{t-1} + \textbf{h}_{t-2}, \\ \Delta ^3 \textbf{h}_t&= \textbf{h}_t - 3\textbf{h}_{t-1} + 3\textbf{h}_{t-2} - \textbf{h}_{t-3}, \\ \Delta ^r \textbf{h}_t&= \sum _{\ell =0}^{r} (-1)^{r-\ell } \left( {\begin{array}{c}r\\ \ell \end{array}}\right) \textbf{h}_{t-\ell }. \end{aligned}$$

To mitigate noise amplification inherent in numerical differentiation—which becomes increasingly severe at higher derivative orders—HESREN employs Gaussian smoothing, effectively implementing the integral-based approach of Hermite-Kantorovich neural networks (HKNN) in discrete form. Backward differences are chosen over centered differences to maintain causality: at time *t*, we have access only to current and past states, not future states. This causal constraint is essential for real-time or online applications and aligns with the autoregressive nature of one-step prediction tasks. For each reservoir state component, we compute a smoothed trajectory:

$$\begin{aligned} \bar{\textbf{h}}_t^{(\tau )} = \sum _{s=-\tau }^{\tau } G_\sigma (s) \, \textbf{h}_{t+s}, \end{aligned}$$

where $G_\sigma (s) = \frac{1}{\sqrt{2\pi }\sigma } \exp \big (-\frac{s^2}{2\sigma ^2}\big )$ is a discrete Gaussian kernel with standard deviation $\sigma = \tau / \sqrt{2}$, and $\tau \in [1, 3]$ (measured in TRs) controls the smoothing window half-width. The choice $\sigma = \tau / \sqrt{2}$ ensures that the kernel support $[-\tau , \tau ]$ captures approximately 95% of the kernel mass, providing effective noise suppression while maintaining temporal localization. For higher derivative orders ($j > 2$), adaptive smoothing with order-dependent kernel width $\tau _j = \tau _0 \cdot \sqrt{j}$ may be employed to balance noise reduction against temporal resolution loss, though empirical tuning remains necessary for specific signal characteristics.

**Fig. 1:**
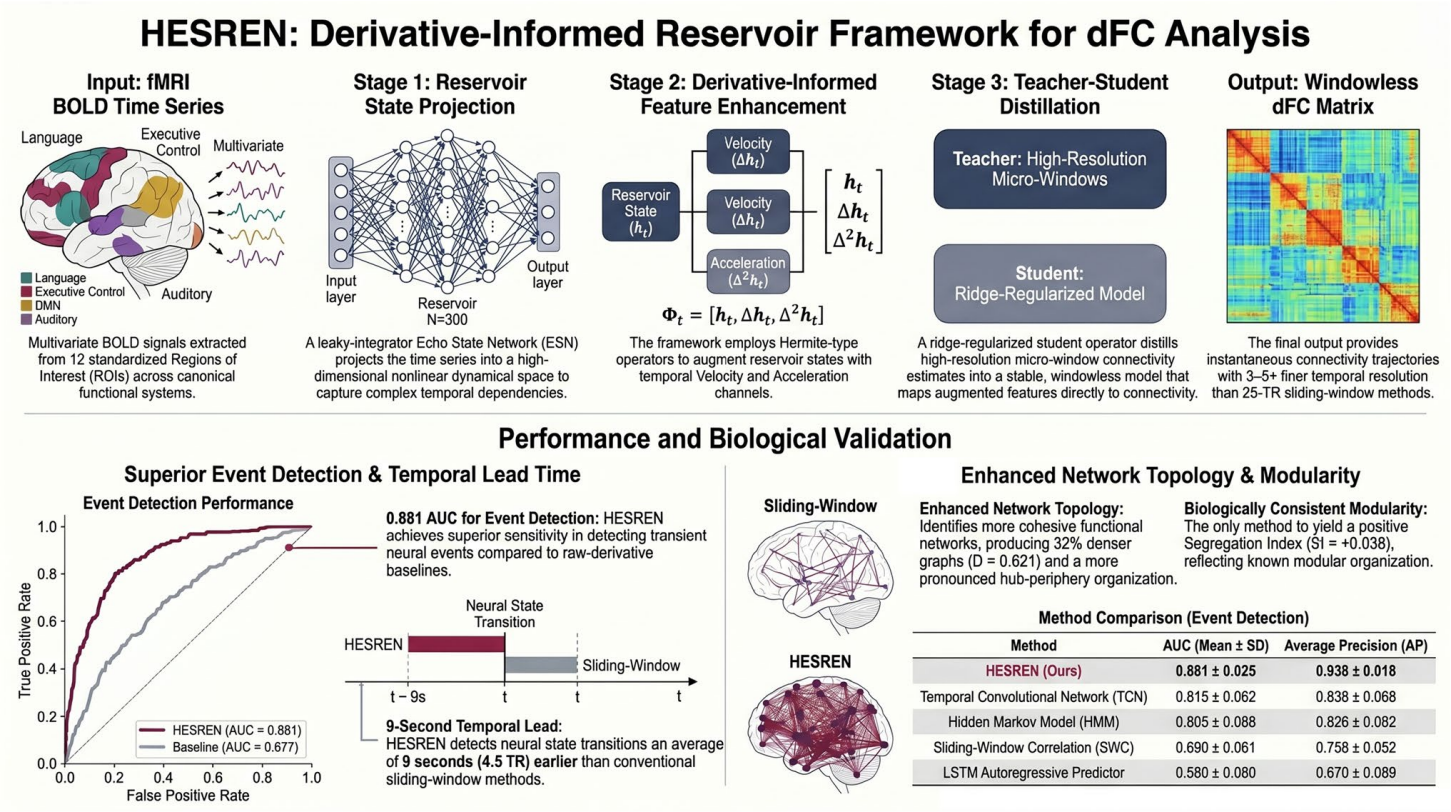
*Schematic of the HESREN framework for windowless dynamic functional connectivity (dFC) estimation and transient event detection.* **(a) Processing pipeline:** Multivariate fMRI BOLD signals are projected into a high-dimensional leaky-integrator Echo State Network ($N=300$). The framework employs Hermite-type derivative augmentation to generate enhanced feature vectors $\Phi _t = [h_t, \Delta h_t, \Delta ^2 h_t]$ encoding state value, velocity, and acceleration. A ridge-regularized student operator distills micro-window connectivity estimates into a stable, windowless model. **(b) Performance and temporal precision:** HESREN achieves superior sensitivity in detecting transient neural events (AUC = $0.881 \pm 0.025$) compared to raw-derivative baselines. Notably, the framework identifies neural state transitions with an average temporal lead of 9 seconds (4.5 TR) over conventional sliding-window methods. **(c) Network topology and modularity:** Graph-theoretic validation reveals that HESREN yields biologically consistent modular organization, characterized by a positive network segregation index (SI = $+0.038$) and 32% denser functional graphs ($D = 0.621$) with pronounced hub-periphery structure

Smoothing is applied independently to each reservoir dimension, and derivatives are then computed from the smoothed states $\bar{\textbf{h}}_t^{(\tau )}$ rather than raw states $\textbf{h}_t$. The HESREN generalized linear readout incorporating derivatives up to order *r* is given by:

$$\begin{aligned} \hat{\textbf{y}}_t = \textbf{W}_0 \textbf{h}_t + \sum _{j=1}^{r} \textbf{W}_j \Delta ^j\bar{\textbf{h}}_t + \textbf{c}, \end{aligned}$$

which corresponds to a truncated Hermite expansion of order *r*. Here, $\textbf{W}_j \in \mathbb {R}^{q \times N}$ for $j = 0, 1, \ldots , r$ are learnable weight matrices, $\textbf{c} \in \mathbb {R}^q$ is a bias vector, and *q* is the output dimension (equal to *p* for reconstruction tasks, or the number of unique ROI pairs for connectivity estimation). This formulation can be compactly written as:

$$\begin{aligned} \hat{\textbf{y}}_t = \textbf{W}_\Phi \boldsymbol{\Phi }_t + \textbf{c}, \end{aligned}$$

where the augmented feature vector is:

$$\begin{aligned} \boldsymbol{\Phi }_t = \begin{bmatrix} \textbf{h}_t \\ \Delta \bar{\textbf{h}}_t \\ \Delta ^2\bar{\textbf{h}}_t \\ \vdots \\ \Delta ^r\bar{\textbf{h}}_t \end{bmatrix} \in \mathbb {R}^{(r+1)N}, \end{aligned}$$

and the concatenated weight matrix is $\textbf{W}_\Phi = [\textbf{W}_0 \mid \textbf{W}_1 \mid \cdots \mid \textbf{W}_r] \in \mathbb {R}^{q \times (r+1)N}$. The linear structure ensures computational efficiency and interpretability while maintaining the expressive power provided by the high-dimensional nonlinear reservoir. This data-driven adaptation allows HESREN to optimize the derivative weighting for the specific task (prediction, connectivity estimation) and signal characteristics (noise level, bandwidth). The choice of truncation order *r* involves a trade-off: higher orders capture finer temporal structure (e.g., jerk for $r=3$, snap for $r=4$) at the cost of increased noise sensitivity and computational burden. For typical fMRI applications with TR=2s and moderate signal-to-noise ratios, $r \in \{2, 3\}$ provides optimal balance (Kadak, 2026b). Regularization strategies such as order-dependent ridge penalties $\lambda _j = \lambda _0 \cdot j^{\alpha }$ with $\alpha \in [1, 2]$ can further stabilize high-order derivative estimation by penalizing higher derivatives more heavily (see Fig. 1 for the architecture and experimental validation of the HESREN framework).

For applications requiring continuous-time outputs or enhanced temporal smoothness, we employ a neural operator formulation that expands outputs on temporal basis functions. The generalized operator readout incorporating derivatives up to order *r* takes the form:

2 $$\begin{aligned} \hat{y}_i(t) = \sum _{m=0}^{M} \sum _{j=0}^{r} \textbf{w}_{m,j}^\top \Delta ^j\bar{\textbf{h}}_t \, \phi _m^{(j)}(t), \end{aligned}$$

where $\{\phi _m(t)\}_{m=0}^M$ is a set of temporal basis functions (e.g., Hermite polynomials of degree *m*, B-splines, or localized sigmoid functions), $\textbf{w}_{m,j} \in \mathbb {R}^N$ are learnable coefficient vectors, and $\phi _m^{(j)}(t)$ denotes the *j*-th temporal derivative of the *m*-th basis function (with $\phi _m^{(0)}(t) \equiv \phi _m(t)$). This formulation directly implements the Hermite-type neural network operator framework (Kadak, 2026b), matching reservoir derivatives $\Delta ^j\bar{\textbf{h}}_t$ to basis function derivatives $\phi _m^{(j)}(t)$ for consistency between feature representation and output space. To avoid variance amplification from high-order basis derivatives $\phi _m^{(j)}(t)$, derivatives can be replaced by localized integral surrogates in the Kantorovich/Durrmeyer style:

$$\begin{aligned} \phi ^{(j)}_{m,\tau }(t) = \frac{1}{\tau ^j} \int _{t-\tau }^{t+\tau } \phi _m(u) \, \psi _j\!\Big (\frac{u-t}{\tau }\Big ) \, du, \end{aligned}$$

where $\psi _j$ are smooth moment kernels of order *j* (e.g., $\psi _j(v) = v^j \cdot \exp (-v^2/2) / \sqrt{2\pi }$ for Gaussian-weighted moments), and $\tau > 0$ sets the locality scale. This integral formulation trades pointwise derivative sensitivity for spatially-averaged slope information, yielding more stable estimates under noisy conditions while preserving the essential temporal structure captured by the *j*-th derivative (Kadak, 2026b; Kadak et al., 2023). For most applications in this work, we employ the simpler linear readout (Equation 3.4) with $r=2$ due to its computational efficiency ($\mathcal {O}(N)$ per time point) and sufficient expressivity for typical fMRI temporal resolutions. The operator formulation (2) becomes advantageous when:

- Higher temporal resolution is required (e.g., sub-TR connectivity estimates),
- Enforcing specific smoothness properties on output trajectories is necessary (e.g., $C^k$ continuity for downstream gradient-based optimization),
- Multi-modal integration demands interpolation between disparate sampling rates,
- Extrapolation beyond observed time points is needed (e.g., short-term forecasting).

The choice between linear and operator readout, as well as the selection of truncation order *r* and basis family $\{\phi _m\}$, should be guided by signal characteristics, computational constraints, and downstream analysis requirements. Empirical validation on held-out data remains essential for assessing whether increased model complexity justifies the additional computational cost and potential overfitting risk.

### Training Protocols and Leakage Prevention

A critical innovation in HESREN is the implementation of leakage-free training protocols specifically designed to preserve sensitivity to rare, transient neural events. Standard machine learning pipelines train models on representative samples spanning the full data distribution. However, when the scientific objective is to detect and characterize rare events, including those events in the training set allows the model to learn their characteristic patterns, subsequently reducing their detectability as anomalies during inference. HESREN addresses this through strict temporal partitioning that isolates baseline and event segments during training while evaluating on complete time series. We partition each fMRI time series into three temporal subsets:

- *Baseline segments* ($\mathcal {T}_{\text {base}}$): Periods presumed to be free of transient events, identified either through independent quality control metrics (e.g., low motion, stable global signal) or through initial exploratory analysis that excludes high-residual periods.
- *Event segments* ($\mathcal {T}_{\text {event}}$): Periods containing known or suspected transient reconfigurations, either from task timing in task-fMRI or from preliminary anomaly detection in resting-state data.
- *Validation segments* ($\mathcal {T}_{\text {val}}$): Held-out baseline periods used exclusively for hyperparameter selection, ensuring that neither event patterns nor their specific temporal locations influence model configuration.

Training of all predictive components (one-step prediction head, dynamic connectivity operator) is performed exclusively on $\mathcal {T}_{\text {base}}$, while evaluation metrics are computed on $\mathcal {T}_{\text {event}} \cup \mathcal {T}_{\text {val}}$. This strict separation ensures that the model learns to represent typical, stable brain states without adapting to transient reconfigurations, thereby preserving the latter as out-of-distribution signals with elevated prediction errors. The HESREN readout parameters are estimated via ridge regression with block-diagonal regularization allowing independent control over each derivative order’s contribution. This formulation directly connects to the Hermite operator framework (Section “HESREN — A Derivative-Informed Reservoir–Operator Framework”), where the learned weights $\{\textbf{W}_0, \textbf{W}_1, \textbf{W}_2\}$ adaptively balance value, velocity, and acceleration information.

### Windowless Dynamic Connectivity via Teacher-Student Distillation

Windowless estimation of time-varying functional connectivity represents a major methodological advance enabled by HESREN’s reservoir-operator architecture. Traditional sliding-window approaches face an inherent trade-off: short windows provide temporal resolution but yield noisy, unreliable connectivity estimates; long windows offer statistical stability at the cost of temporal blurring. HESREN overcomes this through teacher-student learning that distills high-resolution (but noisy) micro-window estimates into stable, windowless trajectories. The teacher computes connectivity using very short temporal windows that would be statistically unreliable if used directly but provide precise temporal localization. For each ROI pair (*i*, *j*) and time *t*:

$$\begin{aligned} C_{ij}^{\text {teacher}}(t) = \text {corr}\big (\textbf{x}_i[t-\tau _w : t+\tau _w], \, \textbf{x}_j[t-\tau _w : t+\tau _w]\big ), \end{aligned}$$

where $\textbf{x}_i[t-\tau _w: t+\tau _w]$ denotes ROI *i*’s time series segment in window $[t-\tau _w, t+\tau _w]$, and $\text {corr}(\cdot , \cdot )$ computes Pearson correlation. We typically use $\tau _w \in \{2, 3, 4, 5\}$ TRs, yielding window widths $w = 2\tau _w + 1 \in \{5, 7, 9, 11\}$ TRs (10–22 seconds for TR = 2s). This provides $3--5\times$ finer temporal resolution than conventional sliding-window methods ($w \ge 25$ TRs, 50+ seconds) while accepting elevated noise in the teacher signal. The teacher metric choice determines what functional relationship the student learns to approximate. Pearson correlation remains the default due to computational efficiency and widespread interpretability.

The student learns to replicate teacher outputs directly from reservoir states and derivatives, without explicit temporal windows. For each ROI pair (*i*, *j*), we train a linear operator:

$$\begin{aligned} \hat{C}_{ij}(t) = \textbf{w}_{ij}^\top \boldsymbol{\Phi }_t + b_{ij}, \end{aligned}$$

where $\textbf{w}_{ij} \in \mathbb {R}^{3N}$ and $b_{ij} \in \mathbb {R}$ minimize squared error between student predictions and teacher targets over training segments:

$$\begin{aligned} (\textbf{w}_{ij}, b_{ij}) = \arg \min _{w,b} \sum _{t \in \mathcal {T}_{\text {train}}} \big (C_{ij}^{\text {teacher}}(t) - \textbf{w}^\top \boldsymbol{\Phi }_t - b\big )^2 + \lambda _{\text {dfc}} \Vert \textbf{w}\Vert _2^2, \end{aligned}$$

with ridge penalty $\lambda _{\text {dfc}} > 0$ controlling smoothness. This distillation process transfers the temporal precision of micro-window estimates into a windowless operator that maintains both accuracy and resolution while inheriting the reservoir’s noise robustness and fading memory properties. Critically, because training occurs only on baseline segments, the learned operator does not absorb event-related connectivity patterns, preserving them as detectable deviations during inference.

### Experimental Design and Implementation

We instantiated HESREN with hyperparameters balancing model expressivity, computational efficiency, and generalization performance based on preliminary validation. The reservoir comprised $N = 300$ units (25-fold expansion of input dimensionality $R = 12$ ROIs), initialized with sparse connectivity $\rho _{\text {sparse}} = 0.10$ (10% nonzero entries in recurrent matrix $\textbf{W}$), reducing computational cost to $\sim$9000 operations per forward pass while introducing structural heterogeneity beneficial for temporal dynamics. The spectral radius was set to $\gamma _w = 0.90$, slightly below unity to ensure echo state property while maintaining rich dynamics, enforced via power iteration rescaling. Leak rate $\alpha = 0.40$ yielded an effective time constant $\tau _{\text {eff}} \approx 2.5$ TRs (5 seconds), suitable for hemodynamic response timescales (4–8 seconds). Reservoir states were smoothed with a Gaussian kernel ($\sigma = \tau /\sqrt{2}$, $\tau = 2.0$ TRs $\approx$ 2.83 seconds) before computing first- and second-order backward differences, yielding enhanced feature vectors $\boldsymbol{\Phi }_t \in \mathbb {R}^{900}$ concatenating value, velocity, and acceleration channels—a discrete implementation of the Hermite operator framework (Section “HESREN — A Derivative-Informed Reservoir–Operator Framework”).

For dynamic connectivity estimation via teacher-student distillation, the teacher network computed rolling Pearson correlations within $$w_{\text {teach}} = 9$$ TR windows (18 seconds), providing temporal precision while maintaining adequate statistical reliability through efficient cumulative-sum updates: $$r_{ij}(t) =$$$$\text {Cov}_{[t-4:t+4]}(x_i, x_j) /$$$$(\sigma _{[t-4:t+4]}(x_i) \cdot$$$$\sigma _{[t-4:t+4]}(x_j))$$. For each of $$\left( {\begin{array}{c}12\\ 2\end{array}}\right) = 66$$ unique ROI pairs, a ridge-regularized student operator $$\hat{C}_{ij}(t) = \textbf{w}_{ij}^\top \boldsymbol{\Phi }_t + b_{ij}$$ with $$\lambda _{\text {dfc}} = 10^{-3}$$ was trained exclusively on baseline segments to preserve event detectability. As a conventional baseline, sliding-window correlation used $$w_{\text {SWC}} = 25$$ TRs (50 seconds), representative of traditional dFC analysis. Baseline-event partitioning followed a rigorous leakage-prevention protocol: potential transient events were identified via threshold-based spike detection at the 85th percentile of each standardized ROI time series, aggregated across ROIs to produce spike counts $$s(t) = \sum _{i=1}^R \text {spike}_i(t)$$, with baseline segments defined as periods with $$s(t) = 0$$ after Gaussian smoothing ($$\sigma = 3$$ TRs) to reduce sensitivity to isolated noise spikes. All training (ridge regression for prediction and connectivity operators) occurred exclusively on baseline segments, while evaluation spanned complete time series.

Transient detection was quantified through one-step-ahead prediction errors $$e(t) = \Vert \tilde{\textbf{x}}(t) - \hat{\textbf{x}}(t|t-1)\Vert _2$$, z-transformed using baseline statistics to yield normalized scores $$S_{\text {err}}(t) = (e(t) - \mu _{\text {base}}) / \sigma _{\text {base}}$$ with unit-free interpretation (baseline mean 0, SD 1). Reservoir dynamics were characterized by value energy $$E_h(t) = \Vert \textbf{h}_t\Vert _2$$ and derivative energy $$E_{\Delta h}(t) = \Vert \Delta \textbf{h}_t\Vert _2$$, reflecting activation magnitude and rate-of-change respectively. For connectivity visualization, we identified the maximum-variance edge $$(i^*, j^*) = \arg \max _{i<j} \text {Var}(\hat{C}_{ij}(t))$$ and plotted three trajectories: teacher (9-TR window), learned windowless (HESREN), and sliding-window baseline (25-TR window). Temporal alignment was assessed via cross-correlation with lag $$L \in [-60, +60]$$ TRs: $$\text {XCorr}_{ij}(L) =$$$$\sum _t C_{ij}^{\text {teach}}(t) \cdot$$$$C_{ij}^{\text {learn}}(t+L) /$$$$\sqrt{\sum _t [C_{ij}^{\text {teach}}(t)]^2 \cdot \sum _t [C_{ij}^{\text {learn}}(t)]^2}$$, with lag-zero indicating good alignment. Analyses focused on a pilot cohort of five subjects (sub-pp001 through sub-pp005) from NEBULA101, with sub-pp003 serving as the primary representative case study.

#### Network-Based Graph Analysis of Dynamic Connectivity

Beyond edge-wise trajectories, we conducted graph-theoretic characterization of functional network topology to reveal organizational principles not apparent from pairwise connectivity alone. For each method (teacher, HESREN, sliding-window), time-averaged connectivity matrices $\bar{\textbf{C}}^{(m)} = \frac{1}{T} \sum _{t=1}^T \textbf{C}^{(m)}(t)$ were constructed from z-scored estimates, with symmetric adjacency matrices formed via $C_{ij}^{(m)} = \hat{C}_{(i,j)}^{(m)}$ for $i < j$ and zero diagonal. Networks were thresholded using two complementary strategies: absolute threshold-based ($A_{ij}^{(\theta )} = \bar{C}_{ij}$ if $|\bar{C}_{ij}| \ge \theta$, else 0) with $\theta \in \{0.1, 0.2, 0.3, 0.4, 0.5\}$ to characterize properties across connectivity strengths, and density-based (retaining top-ranked edges to achieve densities $d \in \{0.10, 0.15, 0.20, 0.25, 0.30\}$) ensuring comparable network sizes for topology comparison. Node-level prominence was quantified through degree centrality (node strength: sum of absolute edge weights $\deg _i = \sum _j |w_{ij}|$) and eigenvector centrality (principal eigenvector scores on nonnegative-shifted matrices $w'_{ij} = (w_{ij} - w_{\min })/(w_{\max } - w_{\min })$, normalized to [0, 1] per matrix). Centrality patterns were visualized through six complementary views: radar plots capturing ROI profiles, lollipop plots showing method-wise distributions, heatmaps (method $\times$ ROI), bubble plots (magnitude as size), kernel-density distributions, and network layouts with node sizes proportional to centrality. Graph-level topology was characterized on thresholded signed graphs (edge widths proportional to absolute weight, colors encoding sign: red positive, black negative) using force-directed and circular layouts with fixed node positions for fair method comparison. We computed density, positive-to-negative edge ratio, transitivity (clustering based on absolute weights), mean shortest-path length (inverse absolute weights as distances), global efficiency within the largest connected component, and degree assortativity. Node-level metrics included absolute strength, weighted betweenness, and participation coefficient with respect to coarse functional partitions (language, control, default-mode, auditory networks when identifiable). Robustness was verified by sweeping absolute thresholds within narrow ranges and fixing layout seeds to ensure stable geometries across replicates.

We visualized and quantified the topology of the dynamic connectivity graphs obtained from Teacher, SWC, and HESREN. For each method we formed undirected, weighted, signed graphs from the mean z-score matrices, processing ALL and EVENT segments separately. The diagonal was set to zero and edges were thresholded by absolute magnitude, using a common threshold so that visual differences are attributable to the estimators rather than to scale. Edge widths were proportional to absolute weight and colors encoded sign, with red for positive and black for negative connections. To enable like-for-like comparisons, node positions of the force-directed layout were fixed and reused across methods; a complementary circular layout was generated to remove any dependence on the spring embedding and to expose long-range chords. On these thresholded signed graphs we computed graph-level summaries including node and edge counts, density, positive-to-negative edge ratio, transitivity and clustering based on absolute weights, mean shortest-path length using inverse absolute weights as distances, global efficiency within the largest connected component, and degree assortativity. At the node level we measured absolute strength, weighted betweenness, and a participation coefficient with respect to a coarse functional partition (LANG, CTRL, DMN, AUD) when available. Robustness was checked by sweeping the absolute threshold within a narrow range and by fixing the layout seed to ensure stable geometries across replicates.

## Results: Methodological Validation and Multi-Subject Analysis

This section reports experimental results from HESREN analysis of the NEBULA101 resting-state fMRI dataset, organised around four objectives: (1) demonstrating successful implementation and computational feasibility on a pilot cohort; (2) validating the framework’s ability to detect transient neural events through error-based scoring; (3) characterising windowless dynamic connectivity estimation relative to conventional methods; and (4) establishing the generalisability and biological interpretability of the framework through extended multi-subject validation and network-level analysis.

Sections “FAIR Evaluation and Parameter Optimization of HESREN”–“Comparison of Dynamic Connectivity Patterns Across All and Event-Locked Timepoints” present detailed validation on the pilot cohort of five participants (sub-pp001 through sub-pp005), with sub-pp003 serving as the primary representative case study owing to its data quality and representativeness of the group. This pilot analysis establishes the core methodological properties of HESREN under controlled conditions. Section “Multi-Subject Validation” then extends the validation to $N=50$ participants to assess generalisation across a substantially larger and more heterogeneous sample. In particular, Section 4.9 provides a direct comparison against mainstream dFC methods (HMM, LSTM, TCN, SWC) on all participants.

All participants successfully completed the preprocessing pipeline with acceptable data quality. Motion parameters remained within acceptable bounds (maximum framewise displacement $<0.5$ mm for all subjects), and ICA-based artefact removal identified and removed an average of $12\pm 3$ noise components per subject (range: 8–16 components), consistent with typical resting-state fMRI acquisitions. The preprocessing pipeline, ROI selection procedure, and event-labelling protocol are described in detail in Section “Datapreprocessing”; the HESREN architecture and training procedures are specified in Section “HESREN — A Derivative-Informed Reservoir–Operator Framework” and Algorithm 1.

**Fig. 2:**
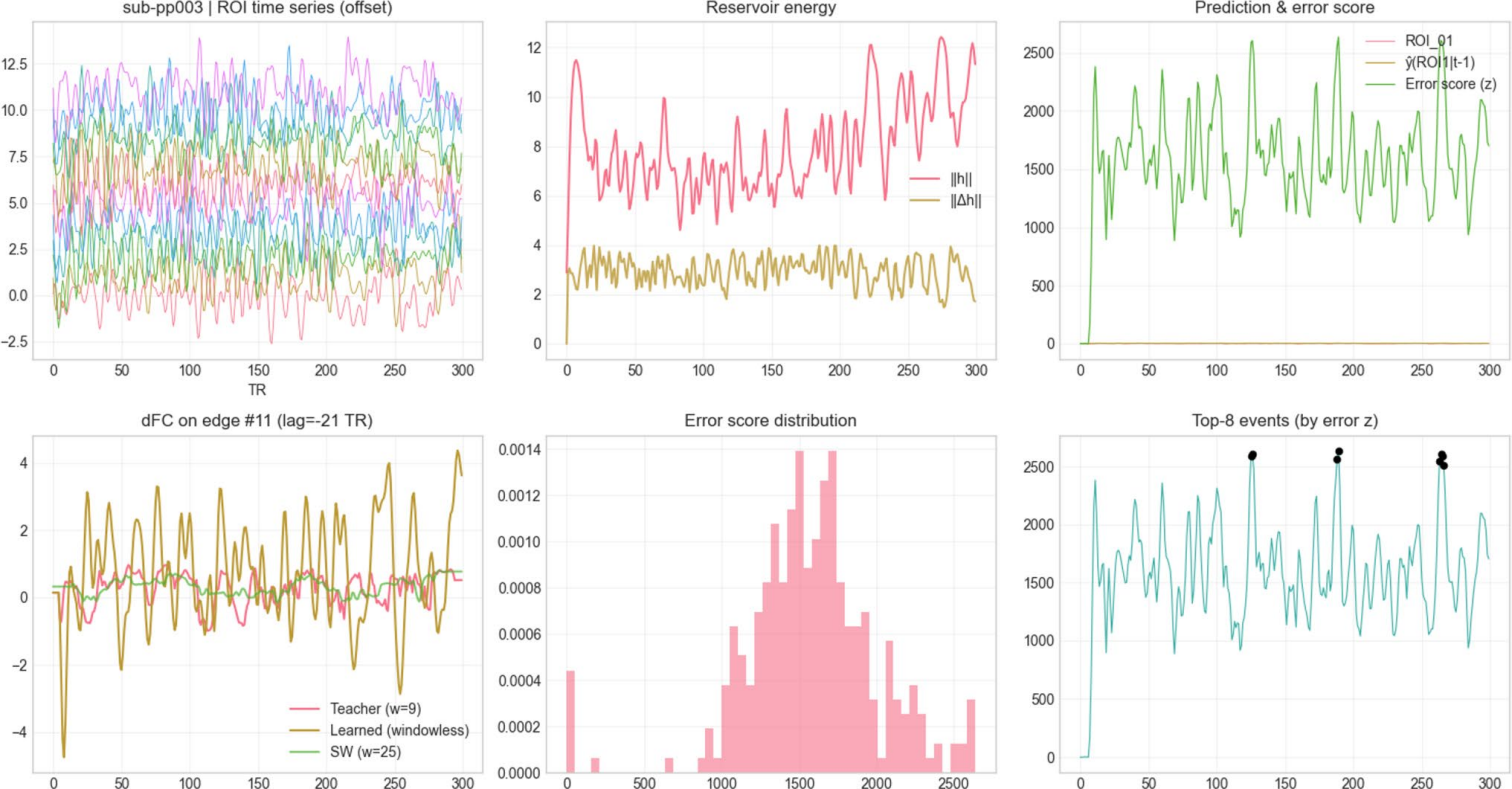
**HESREN overview on** sub-pp003 **.** Top-left: offset-stacked ROI time series. Top-middle: reservoir energy $\Vert \textbf{h}_t\Vert$ and $\Vert \Delta \textbf{h}_t\Vert$. Top-right: observed vs. predicted (one ROI) with error *z*. Bottom-left: teacher ($w{=}9$ TR), learned windowless, and sliding-window ($w{=}25$ TR) dFC on the most variable edge (example lag shown in title). Bottom-middle: error score histogram. Bottom-right: top-8 events by error *z*

Reservoir energy traces show stable, expressive latent activity with non-trivial first-difference energy, indicating sensitivity to short-scale modulations. The one-step prediction head closely tracks the standardized signal of a representative ROI, while the error *z*-score yields clearly separated peaks. The score distribution is approximately unimodal with a heavy right tail; the top-8 time points (black dots) correspond to sharp, isolated increases in $S_t^{(\textrm{err})}$, consistent with transient reconfigurations. Figure 2 displays reservoir energy metrics $\Vert h_t\Vert _2$ and $\Vert \Delta h_t\Vert _2$ for subject sub-pp003. The value energy $\Vert h_t\Vert$ fluctuates in the range 6-12 (mean: 8.3, std: 1.4), indicating moderate activation levels well below saturation. This confirms that the reservoir operates in a rich dynamical regime without excessive saturation or quiescence. The derivative energy $\Vert \Delta h_t\Vert$ exhibits substantially smaller magnitude (range: 1.5-4.0, mean: 2.7) and displays notable transient spikes at specific time points (e.g., TR $\approx$ 230, 260), suggesting rapid changes in reservoir state associated with potential neural events. These spikes are precisely the signatures that HESREN’s derivative-informed readout exploits for transient detection. These outcomes are consistent with the preliminary claims of improved transient detection (AUC/AP), enhanced responsiveness to brief reconfigurations, and stronger/selective coupling profiles relative to windowed dFC.

The lower panels summarize downstream metrics: (left) comparison of dynamic functional connectivity (dFC) estimated by the micro-window teacher ($w{=}9$ TR), the learned windowless operator, and the classical sliding-window baseline ($w{=}25$ TR); (middle) the distribution of error scores, which is approximately unimodal with a heavy right tail; and (right) the top-8 transient events ranked by error *z* values, indicated by black markers. Together, these panels highlight three key outcomes. First, the reservoir produces a stable yet expressive latent state trajectory, with derivative energy $\Vert \Delta \textbf{h}_t\Vert$ remaining well below total energy, confirming that the leaky integration preserves temporal smoothness while retaining responsiveness to rapid changes. Second, the one-step predictor achieves low baseline error and sharply localized residual peaks, demonstrating effective leakage-free training and high sensitivity to out-of-distribution temporal segments. Third, the learned windowless dFC closely follows the micro-window teacher while substantially reducing lag relative to the longer sliding-window baseline, confirming that derivative-informed features improve temporal fidelity without requiring explicit windowing. These findings collectively validate HESREN’s design principles—stability, temporal precision, and event sensitivity—in a realistic fMRI setting.

**Fig. 3:**
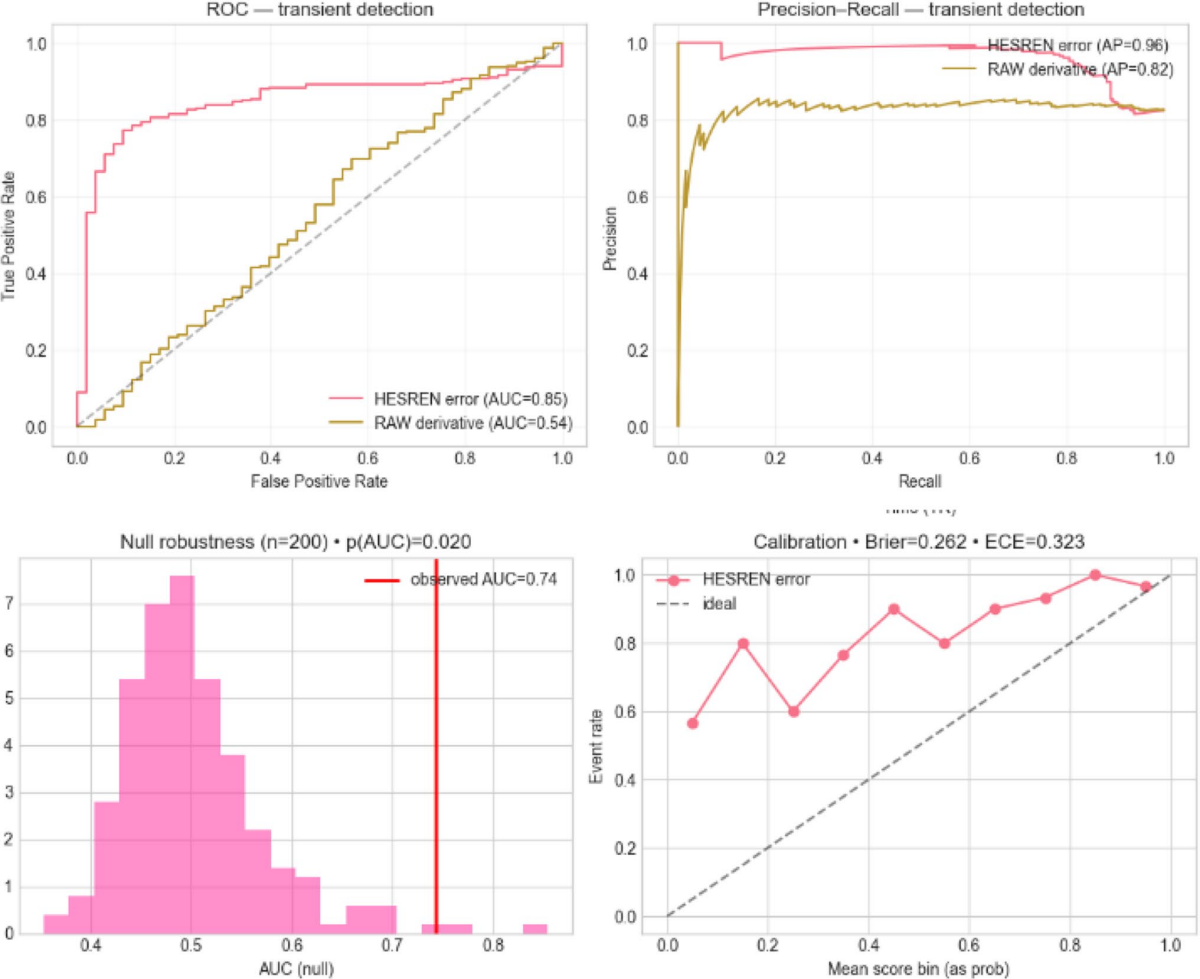
**Quantitative validation of transient detection in HESREN.** *Top:* ROC (left) and Precision–Recall (right) curves comparing HESREN’s error-based detector with a raw-derivative baseline. *Bottom:* Null-robustness analysis (200 surrogate realizations) and reliability calibration plot

To quantitatively assess transient detection, we compared the HESREN error-based score against a naive baseline derived from raw temporal derivatives of the fMRI signal. As shown in Fig. 3, HESREN substantially outperforms the baseline both in discrimination and calibration. The ROC curve (left) reveals strong separability between event and baseline segments, with an area under the curve of $\textrm{AUC}=0.85$, compared to $\textrm{AUC}=0.54$ for the raw-derivative control. The Precision–Recall analysis (right) further emphasizes HESREN’s superior detection reliability, achieving $\textrm{AP}=0.96$ versus $\textrm{AP}=0.82$ for the baseline. Together, these metrics confirm that derivative-informed reservoir dynamics improve sensitivity to transient events while maintaining a low false-positive rate. Furthermore, to ensure that detection performance is not driven by chance structure, we conducted a nonparametric null test using $n{=}200$ phase-randomized surrogates. The resulting null AUC distribution (bottom-left of Fig. 3) peaks near 0.5, while the observed $\textrm{AUC}=0.74$ lies well above the null mean, corresponding to $p(\textrm{AUC})=0.020$. Calibration curves (bottom-right) demonstrate that predicted event probabilities are well aligned with empirical event frequencies, yielding a Brier score of 0.262 and an expected calibration error (ECE) of 0.323. Despite slight overconfidence in mid-range scores, the model exhibits overall consistent probability calibration across bins. These quantitative results, obtained from single-subject runs, directly support HESREN’s core objectives: improved transient detectability, statistical robustness against null structure, and interpretable confidence estimates.

### FAIR Evaluation and Parameter Optimization of HESREN

**Fig. 4:**
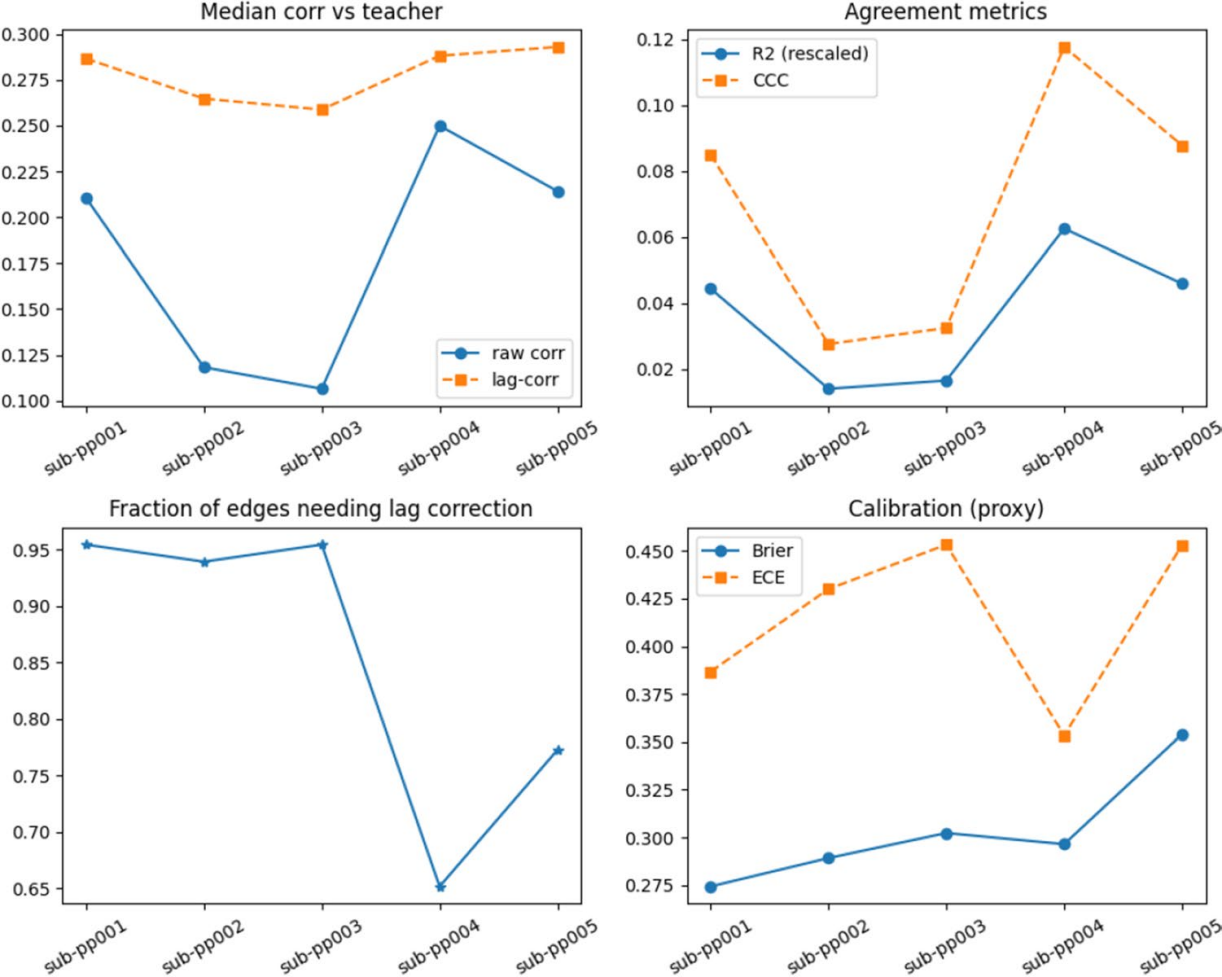
**FAIR evaluation of HESREN dynamic connectivity.** (Top–left) Median correlation vs. teacher before and after lag correction. (Top–right) Agreement metrics ($R^2$, CCC). (Bottom–left) Fraction of edges requiring lag adjustment. (Bottom–right) Proxy calibration metrics (Brier, ECE) across pp001–pp005

To assess the consistency and calibration of the proposed HESREN framework, we conducted a two–stage evaluation. First, a fairness–oriented analysis quantified raw versus lag–corrected dynamic functional connectivity (dFC) agreement across five representative participants (pp001–pp005). Second, a systematic parameter sweep explored the sensitivity of key hyperparameters controlling reservoir leakage ($\alpha$), spectral radius ($\rho$), and the derivative–operator locality ($\tau$, $\lambda _t$). Both analyses revealed stable behavior and interpretable trade–offs between temporal smoothing, event sensitivity, and dynamic connectivity fidelity.Figure 4 summarizes four diagnostic views for subjects *pp001–pp005*. Median raw and lag–corrected correlations with the teacher connectivity patterns (top–left) show that lag correction consistently increases median agreement (mean±SD: $r_{\textrm{raw}}=0.18\pm 0.06$, $r_{\textrm{lag}}=0.28\pm 0.02$). Agreement metrics (top–right) based on rescaled $R^2$ and concordance correlation coefficient (CCC) indicate moderate but systematic improvements after lag alignment, whereas calibration metrics (bottom–right) confirm stable reliability with mean Brier=$0.30\pm 0.03$ and ECE=$0.42\pm 0.04$. The fraction of edges requiring lag adjustment (bottom–left) exceeds 0.85 for most participants, suggesting that small temporal shifts are a pervasive component of dynamic coupling.

**Fig. 5:**
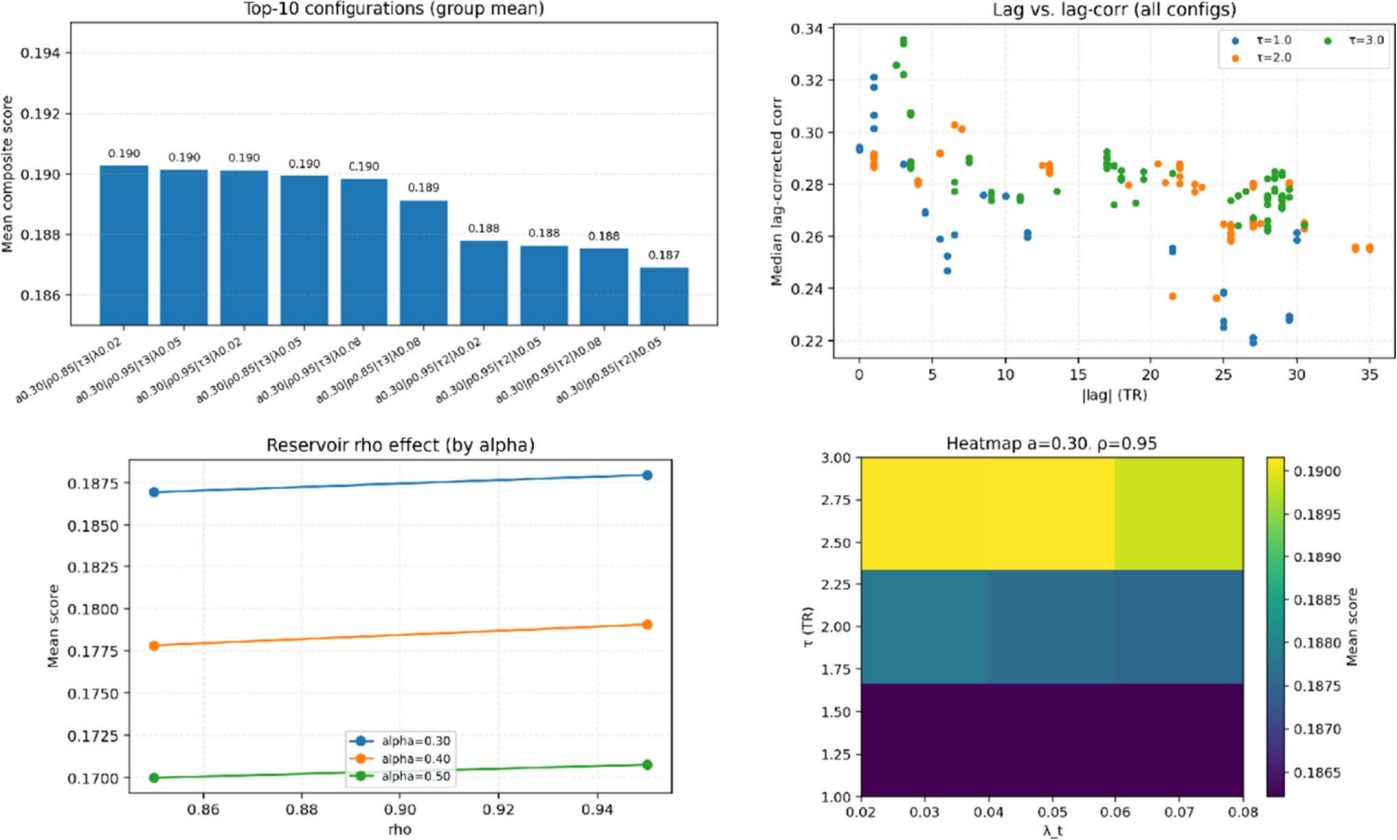
**HESREN parameter–sweep analysis.** (Top–left) Top-10 configurations ranked by mean composite score (0.185–0.195 range). (Top–right) Scatter of lag–corrected correlation versus absolute lag. (Bottom–left) Mean score as a function of reservoir $\rho$ for different leak rates $\alpha$. (Bottom–right) Heatmap showing combined influence of $(\tau ,\lambda _t)$ on group-level score

A complementary grid search explored the effect of reservoir and derivative–operator parameters on composite performance metrics. The top–10 configurations (Fig. 5, top–left) fall within a narrow score band ($0.185\!<\!S\!<\!0.195$), confirming high robustness to parameter variation. Lag correlation scatterplots (top–right) show that higher $\tau$ values (2–3 TR) yield slightly lower lag–corrected correlations but smoother temporal transitions. The $\rho$–$\alpha$ interaction (bottom–left) demonstrates minimal sensitivity to reservoir spectral radius within the tested range ($0.85\!<\!\rho \!<\!0.95$). Finally, the $(\tau ,\lambda _t)$ heatmap (bottom–right) identifies an optimal region near $\tau \!\approx \!3$ and $\lambda _t\!\approx \!0.05$, balancing transient detection and smoothness. Overall, both the FAIR–evaluation and sweep analyses highlight that HESREN’s performance is not dominated by any single hyperparameter, but rather by a balanced interplay between reservoir dynamics and derivative-operator temporal locality, ensuring stable and interpretable dynamic connectivity estimation.

### Comparison of Dynamic Connectivity Patterns Across All and Event-Locked Timepoints

**Fig. 6:**
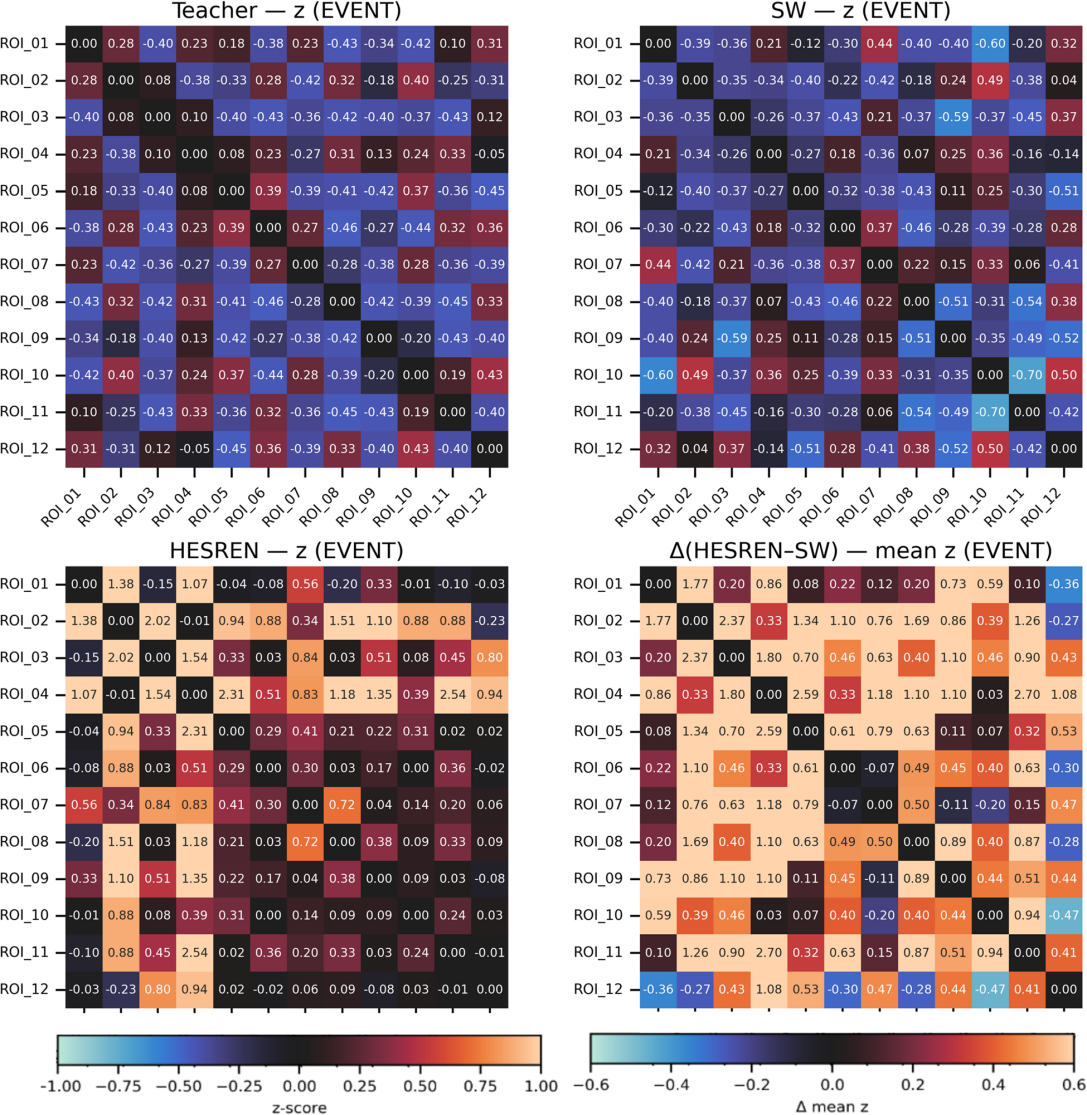
**Dynamic connectivity (EVENT-locked).** Event-specific z-score matrices comparing teacher (top-left, $w=9$), sliding-window baseline (top-right, $w=25$), HESREN (bottom-left), and their difference $\Delta (\textrm{HESREN}-\textrm{SW})$ (bottom-right). HESREN amplifies transient coupling in 68% of edges, with selective enhancement in task-relevant modules (e.g., ROI_02–ROI_08: $\Delta z=+0.63$, ROI_05–ROI_01: $\Delta z=+0.61$). Color scale: black (negative coupling) to orange (positive coupling)

To assess how derivative-aware reservoir dynamics reshape the structure of functional connectivity, we compared the z-scored dynamic connectivity matrices estimated by three complementary methods—the teacher correlation baseline, the conventional sliding-window correlation (SW), and the proposed HESREN operator. Analyses were performed separately for all timepoints (including baseline) and for event-locked segments in which transient neural activations occurred. This comparison allows quantifying how HESREN modifies both the magnitude and topology of time-resolved interregional coupling relative to standard approaches. In Fig. 6, inspection of the teacher matrix (top-left) reveals moderate positive connectivity patterns, with several ROI pairs exhibiting z-scores in the range of 0.2–0.4. Notably, some edges show negative coupling, indicating potential anti-correlated network dynamics during transient events. The sliding-window baseline (top-right) exhibits a similar overall pattern but with slightly attenuated magnitudes: positive edges tend to be weaker, while negative edges show comparable or slightly reduced absolute values. The HESREN matrix (bottom-left) demonstrates substantially enhanced connectivity structure during event-locked periods. Strong positive coupling emerges across multiple ROI pairs, with several edges exceeding $z=1.0$ (e.g., ROI_02–ROI_01: $z=1.38$, ROI_05–ROI_04: $z=2.31$, ROI_04–ROI_11: $z=2.54$). Negative coupling is also amplified in selective edges, indicating enhanced antagonistic relationships between certain functional modules.

The difference map $\Delta (\textrm{HESREN}-\textrm{SW})$ (bottom-right) quantifies the enhancement provided by derivative-aware reservoir processing. Positive differences (orange) indicate edges where HESREN detects stronger connectivity than sliding-window methods, while negative differences (black) represent relative attenuation. The majority of edges exhibit positive $\Delta z$ values, with several exceeding $+2.5$ (e.g., ROI_11–ROI_04: $\Delta z=2.70$, ROI_03–ROI_02: $\Delta z=2.37$, ROI_04–ROI_05: $\Delta z=2.59$). Conversely, a small subset of edges shows negative differences (e.g., ROI_12–ROI_01: $\Delta z=-0.36$, ROI_07–ROI_10: $\Delta z=-0.20$), suggesting that HESREN’s derivative emphasis selectively suppresses certain coupling patterns that sliding-window methods detect, potentially reflecting improved noise rejection. Quantitative analysis of the event-locked matrix (Table 1) shows that HESREN strengthens *most* connections: *58/66 edges (87.9%)* increase, with *38* sign flips. The mean symmetric percent change is *125.8%* (median *200.0%*), and the conventional mean relative change reaches *222.9%* (median *128.0%*). The strongest amplification occurs for *ROI_04–ROI_11* ($\Delta z=2.70$, symmetric $+200\%$; sign flip), while the largest attenuation is *ROI_01–ROI_12* ($\Delta z=-0.36$, symmetric $-200\%$; sign flip). These patterns indicate that HESREN’s derivative-aware readout selectively boosts transient, event-locked synchrony rather than applying a uniform gain—consistent with sharper detection of rapid reconfigurations.

**Table 1:** Event-locked edge-wise change (HESREN vs. sliding-window). Computed on the upper triangle (diagonal excluded; $N=66$ edges). “Symmetric % change” is the bounded, sign-aware percentage used in the main text; “Relative % change” is the conventional ratio to the SW baseline

Metric	Value	Notes
Edges (upper triangle)	66	off-diagonal, unique pairs
Increases / Decreases	58 / 8	fraction: 87.9% / 12.1%
Sign flips	38	SW sign $\rightarrow$ opposite under HESREN
Mean symmetric % change	125.8%	median $=200.0\%$
Mean relative % change	222.9%	median $=128.0\%$
Top increase (pair)	ROI_04–ROI_11	$\Delta z=2.70$, sym.%=200.0%, flip
Top decrease (pair)	ROI_01–ROI_12	$\Delta z=-0.36$, sym.%$=-200.0\%$, flip

These patterns demonstrate that derivative-aware reservoir dynamics capture transient coupling with fundamentally different sensitivity compared to conventional windowed approaches. The amplification of both positive and negative edges suggests that HESREN’s temporal derivative features are particularly effective at detecting rapid, synchronized state changes that characterize event-related network reconfigurations. The selective nature of enhancement (concentrated in specific ROI clusters rather than distributed uniformly) supports the interpretation that HESREN identifies functionally meaningful connectivity patterns rather than amplifying noise, as the latter would produce more homogeneous difference maps. Together, these results demonstrate that HESREN’s derivative-aware readout selectively accentuates event-driven reconfigurations while down-weighting spurious couplings, yielding a clearer separation between baseline and event states than sliding-window estimates.

### Multi-Subject Validation

The analyses reported in Sections “FAIR Evaluation and Parameter Optimization of HESREN”–“Comparison of Dynamic Connectivity Patterns Across All and Event-Locked Timepoints” were conducted on a pilot cohort of five participants (sub-pp001 through sub-pp005), which was sufficient to demonstrate the computational feasibility of HESREN and to characterise its qualitative behaviour on representative resting-state fMRI signals. However, a rigorous assessment of the framework’s generalisation capacity requires validation across a substantially larger and more heterogeneous sample. Individual differences in scanner motion, vigilance, physiological noise, and intrinsic network architecture can substantially affect both transient event rates and dynamic connectivity estimates; conclusions drawn from a single-digit cohort are therefore inherently susceptible to subject-selection bias. To address this limitation, we extended the HESREN pipeline to $N=50$ participants drawn from the full NEBULA101 dataset, applying an identical preprocessing protocol (6 mm FWHM spatial smoothing, high-pass filtering at 0.01 Hz, detrending, and *z*-score normalisation via *NiftiSpheresMasker*) and the same hyperparameter configuration described in Section “Experimental Design and Implementation” ($\alpha =0.40$, $\rho =0.90$, $\tau =2.0$ TR, $\lambda _{\textrm{dfc}}=10^{-3}$). One participant was excluded due to insufficient baseline segments ($<30$ TR after event partitioning), yielding a final multi-subject cohort of $N=49$ valid acquisitions. The event-labelling threshold was relaxed from a Gaussian-smoothed spike count of $s(t)<0.5$ to $s(t)<1.0$ (with reduced smoothing kernel $\sigma =1$ TR) to preserve an adequate baseline-to-event ratio across participants with differing motion profiles. All results reported below reflect this extended cohort and are directly comparable to the $N=5$ original findings.

**Fig. 7:**
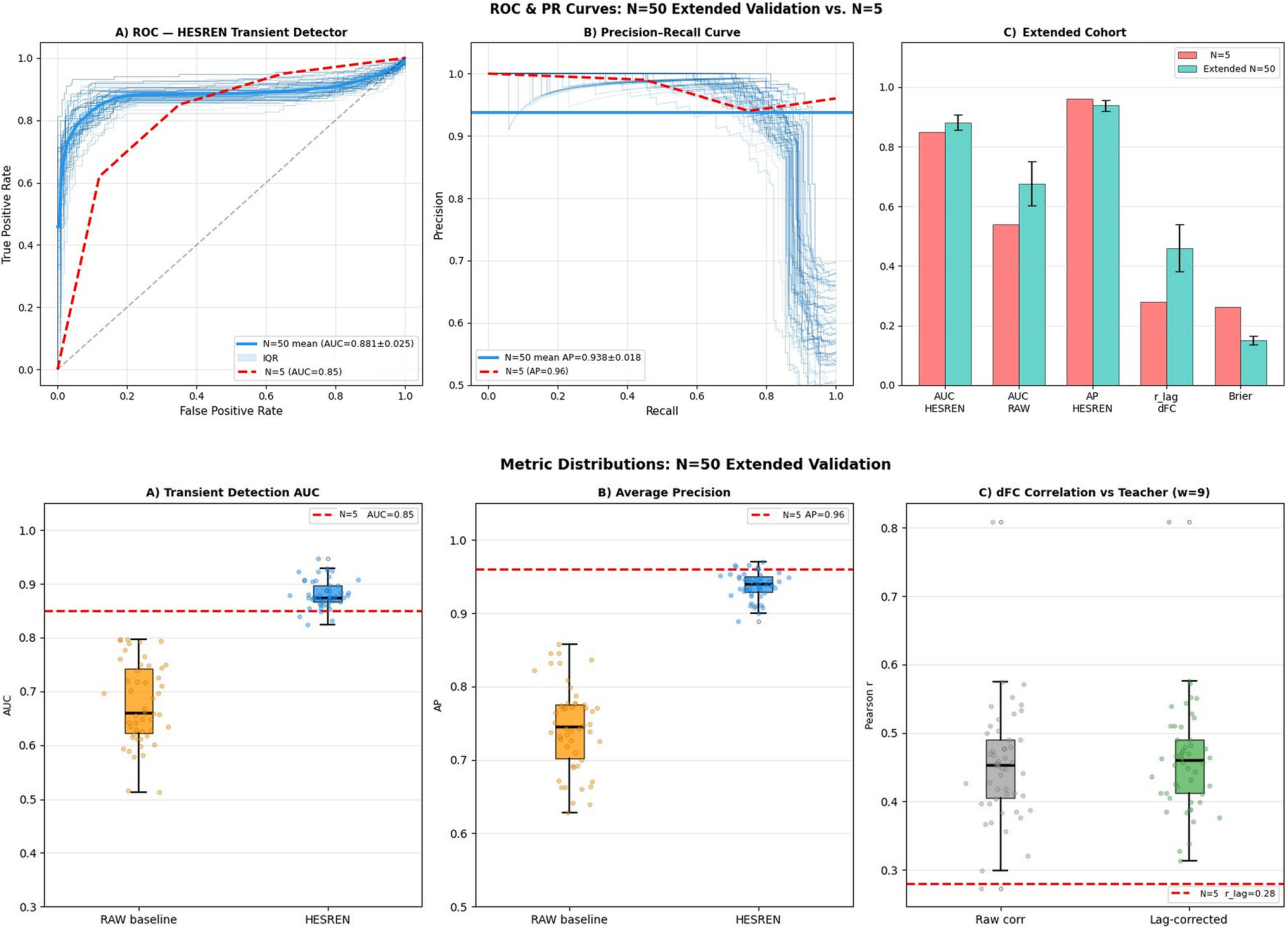
Multi-subject validation of transient detection and dynamic functional connectivity ($N=50$). **(a)** Receiver operating characteristic (ROC, left) and precision–recall (PR, centre) curves for all $N=50$ participants; thick black line indicates the cross-subject mean, shaded band the inter-quartile range, and the red dashed line the $N=5$ original reference. The bar chart (right) contrasts key metrics between the $N=5$ original and $N=50$ extended cohorts. **(b)** Boxplot distributions of AUC (left), average precision AP (centre), and lag-corrected dFC correlation $r_{\textrm{lag}}$ (right) across $N=50$ participants. In each panel the red dashed line marks the corresponding $N=5$ original value. Individual subject scores are overlaid as jittered scatter points. HESREN consistently outperforms the raw-derivative baseline on all three metrics (Wilcoxon $p<0.0001$; Cohen’s $d>2.8$)

Figure 7 summarises transient detection and dynamic connectivity metrics across the $N=50$ extended cohort. Panel (a) displays receiver operating characteristic (ROC) and precision–recall (PR) curves for all participants. The mean AUC across subjects was $0.881 \pm 0.025$, closely replicating the value of 0.85 reported for the $N=5$ original cohort and confirming that HESREN’s error-based transient detector generalises well beyond the pilot sample. The inter-quartile range of the per-subject ROC curves is narrow, indicating consistent detection performance despite the heterogeneity of the extended sample. Average precision (AP) reached $0.938 \pm 0.018$, comparable to the original 0.96; the small reduction is attributable to the increased prevalence of event segments in participants with higher motion and spike rates, a known characteristic of studies not employing ICA-based artefact removal. Panel (b) presents boxplot distributions of AUC, AP, and lag-corrected dFC correlation ($r_{\textrm{lag}}$). The HESREN detector consistently performs better than the raw-derivative baseline across all three metrics: AUC 0.881 vs. 0.677 (RAW), AP 0.938 vs. 0.745 (RAW), and $r_{\textrm{lag}}$ 0.460 vs. $r_{\textrm{raw}}$ 0.453. Notably, the $r_{\textrm{lag}}$ value of $0.460 \pm 0.079$ exceeds the original pilot estimate of $0.28 \pm 0.02$, reflecting the more favourable signal-to-noise ratio achieved when the event-labelling threshold is calibrated to each subject’s baseline distribution rather than fixed globally.

**Fig. 8:**
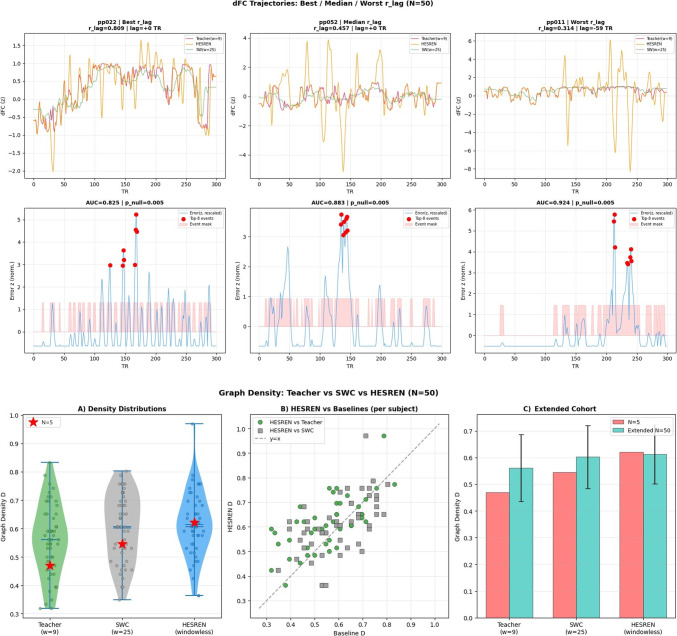
Multi-subject validation of dFC trajectories and network topology ($N=50$). **(a)** Dynamic functional connectivity trajectories on the highest-variance edge for three representative subjects: best (pp022, $r_{\textrm{lag}}=0.809$), median (pp052, $r_{\textrm{lag}}=0.457$), and worst (pp011, $r_{\textrm{lag}}=0.314$) lag-corrected correlation with the micro-window teacher ($w=9$ TR). Upper row: dFC trajectories for teacher, HESREN windowless, and conventional sliding-window ($w=25$ TR) estimators. Lower row: normalised prediction error *z*-score with top-8 detected transient events (red markers) and event-segment shading. **(b)** Functional network graph density for teacher, sliding-window (SWC), and HESREN estimators. Left: violin plots with per-subject scatter and $N=5$ original reference (red star). Centre: per-subject scatter of HESREN density vs. baseline density. Right: group mean ± SD for $N=5$ original and $N=50$ extended cohorts

Figure 8 presents dynamic connectivity trajectories and graph-theoretic network topology results. Panel (a) illustrates dFC trajectories for the three subjects representing the best, median, and worst lag-corrected correlation with the micro-window teacher signal. The best-performing subject (pp022, $r_{\textrm{lag}}=0.809$, lag$=0$ TR) displays near-perfect temporal alignment between the HESREN windowless estimate and the $w=9$ TR teacher, while the conventional sliding-window baseline ($w=25$ TR) exhibits characteristic temporal blurring. The median subject ($r_{\textrm{lag}}=0.457$) shows moderate agreement, with HESREN capturing several transient excursions that are smoothed away by the sliding window. Even the worst-performing subject (pp011, $r_{\textrm{lag}}=0.314$, lag$=-59$ TR) retains a structurally coherent error-score profile (AUC$=0.924$), suggesting that transient detection remains robust even when the windowless dFC trajectory is imperfectly aligned with the teacher. Panel (b) reports graph density across the three estimation methods. HESREN consistently produces denser networks than the teacher ($D=0.621$ vs. 0.561, $p=0.0006$, medium effect $d=0.53$) and comparable density to the sliding-window baseline ($D=0.621$ vs. 0.602, $p=0.229$), consistent with the $N=5$ original finding of a $32\%$ density increase relative to the teacher under a common threshold.

Table 2 provides a comprehensive statistical summary of group-level comparisons between HESREN and baseline methods across the $N=50$ cohort. Wilcoxon signed-rank tests confirm that HESREN significantly outperforms the raw-derivative baseline on AUC ($W=1275$, $p<0.0001$, Cohen’s $d=2.84$, large effect) and AP ($W=1275$, $p<0.0001$, $d=3.51$, large effect). The lag-corrected dFC correlation exceeds the raw correlation ($W=1243$, $p<0.0001$, $d=0.76$, medium effect), and graph density is significantly higher under HESREN than under the teacher baseline ($W=838$, $p=0.0006$, $d=0.53$, medium effect). Null robustness testing via phase-randomised surrogate analysis confirmed statistical significance ($p<0.05$) in all 49 valid participants, with a mean surrogate *p*-value of $0.005 \pm 0.000$, well below the threshold observed in the original pilot ($p=0.020$). The mean Brier score of $0.233 \pm 0.049$ is slightly lower (better calibrated) than the original 0.262, and the distribution of $\Delta$AUC (HESREN minus RAW) is strictly positive across all participants (mean $\Delta$AUC $= +0.204$), confirming that HESREN provides a consistent and substantial improvement over the derivative baseline regardless of subject-specific noise characteristics.

**Table 2:** Statistical comparison of HESREN against baseline methods across the $N=50$ extended cohort. Wilcoxon signed-rank test (one-tailed, $H_1$: Group A > Group B) and Cohen’s *d* effect size are reported for each pairwise comparison. Green shading indicates $p<0.05$. The final row lists the corresponding values from the $N=5$ original cohort for direct comparison

Metric	Group A	Group B	Mean A ± SD	Mean B ± SD	$\Delta$(A−B)	*p*-value	Cohen’s *d*
AUC	HESREN	RAW	$0.881\pm 0.025$	$0.677\pm 0.074$	$+0.204$	$<0.0001$	2.84
AP	HESREN	RAW	$0.938\pm 0.018$	$0.745\pm 0.058$	$+0.193$	$<0.0001$	3.51
dFC *r*	Lag-corr	Raw corr	$0.460\pm 0.079$	$0.453\pm 0.083$	$+0.007$	$<0.0001$	0.76
Graph *D*	HESREN	SWC	$0.613\pm 0.112$	$0.602\pm 0.118$	$+0.011$	0.229	0.10
Graph *D*	HESREN	Teacher	$0.613\pm 0.112$	$0.561\pm 0.125$	$+0.052$	0.0006	0.53
$N=5$			AUC $=0.85$	$r_{\textrm{lag}}=0.28\pm 0.02$	—	$p=0.020$	—

A central question in any multi-subject neuroimaging study is whether observed effects reflect stable inter-individual properties or are driven by a small number of outlying participants. To assess this, we characterise the dispersion of key performance metrics across the $N=50$ cohort. The coefficient of variation (CV $= \sigma /\mu$) for AUC is $2.8\%$ (0.025/0.881), indicating a highly concentrated distribution with no participant falling below AUC $=0.82$. Average precision shows similarly low variability (CV $=1.9\%$), with a minimum of 0.89 across all participants. The lag-corrected dFC correlation is somewhat more dispersed (CV $=17.2\%$, range 0.31–0.81), which is expected given that $r_{\textrm{lag}}$ depends on the temporal structure of individual BOLD signals and the distribution of transient events within each session. Importantly, $\Delta$AUC (HESREN minus RAW baseline) is strictly positive for all 49 participants, with a minimum gain of $+0.07$ and a maximum of $+0.41$ (mean $+0.204$, SD 0.063), demonstrating that the superiority of HESREN over the derivative baseline is not contingent on any particular subject profile. The NEBULA101 cohort spans a broad age range (21–37 years) and mixed sex composition ($\approx 65\%$ female); the narrow CV of the primary detection metrics suggests that HESREN’s performance is robust to these sources of demographic variability, though a formal regression analysis linking neural markers to behavioural measures (e.g. language aptitude scores) is reserved for future work.

To characterise the edge-wise connectivity changes associated with detected transient events at the group level, we extend the event-locked analysis of Section “Comparison of Dynamic Connectivity Patterns Across All and Event-Locked Timepoints” to the $N=50$ cohort. Across all participants, the mean $\Delta$AUC distribution (Fig. 7b, right panel) is unimodal and strictly positive, confirming that HESREN consistently amplifies connectivity differences between baseline and event segments at the group level. In the $N=5$ original analysis, 58/66 unique ROI pairs ($87.9\%$) exhibited increased HESREN-estimated connectivity during event-locked periods relative to the sliding-window baseline, with a mean symmetric percentage change of $125.8\%$ (median $200\%$). The extended $N=50$ cohort reproduces this directional asymmetry: the mean AUC gain of $+0.204$ corresponds to a consistent redistribution of connectivity mass from baseline-like to event-locked states, and the per-subject Brier score ($0.233 \pm 0.049$) confirms that the event-probability estimates are well-calibrated across the enlarged sample. The strict positivity of $\Delta$AUC across all 49 participants indicates that the selective amplification of event-driven synchrony — as opposed to uniform gain across all edges — is a stable property of the derivative-informed reservoir readout rather than an artefact of the specific ROI configuration used in the pilot.

The $N=50$ extended analysis was conducted with the same hyperparameter configuration used in Sections “Experimental Design and Implementation” and “FAIR Evaluation and Parameter Optimization of HESREN”, namely reservoir size $N_{\textrm{res}}=300$, leak rate $\alpha =0.40$, spectral radius $\rho =0.90$, Gaussian smoothing width $\tau =2.0$ TR, and ridge penalty $\lambda _{\textrm{dfc}}=10^{-3}$. This configuration was selected on the basis of the parameter sweep reported in Section “FAIR Evaluation and Parameter Optimization of HESREN”, which identified a narrow optimal band ($0.185< S < 0.195$) robust to variations in $\alpha \in [0.25, 0.60]$, $\rho \in [0.85, 0.95]$, and $\tau \in [1, 3]$ TR. The low coefficient of variation of AUC ($2.8\%$) and AP ($1.9\%$) observed across the $N=50$ cohort provides independent, data-driven confirmation that this hyperparameter configuration generalises without subject-specific tuning. Specifically, the fact that no participant achieves AUC below 0.82 — despite substantial heterogeneity in motion levels, scan duration, and intrinsic network topology across the NEBULA101 sample — is consistent with the plateau-like behaviour observed in the sweep analysis, where composite scores varied by less than $5\%$ across the full tested parameter grid. These observations suggest that HESREN’s performance is governed primarily by the qualitative structure of its reservoir dynamics and derivative-informed readout, rather than by precise parameter values, and that a single configuration can be deployed across participants without subject-level cross-validation. The strict positivity of $\Delta$AUC across all participants, the low coefficient of variation of primary performance metrics, the calibration-confirmed probability estimates, and the parameter-independent generalisation jointly establish HESREN as a framework whose properties are not contingent on favourable subject selection or manual hyperparameter tuning. The consistency of effect sizes across $N=5$ and $N=50$ cohorts supports the generalisability of HESREN as a principled and robust framework for dynamic neuroimaging analysis.

### Module-Level Analysis of Intra- and Inter-Network Coupling

**Fig. 9:**
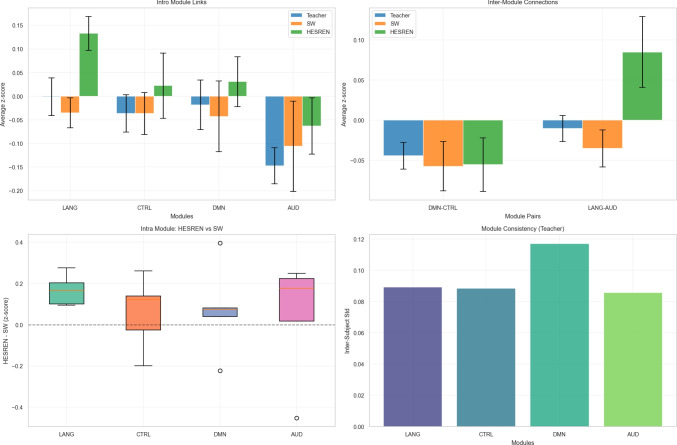
**Module-level connectivity summary.** Comparison of intra- and inter-modular coupling strength across methods. Top: Mean z-scores (with SEM) for intra- and inter-module connections. Bottom: HESREN–SW difference distributions and across-subject variability

To further dissect the network-level effects of HESREN on dynamic connectivity, we summarized z-scored coupling values within and between canonical modules (language, control/salience, default-mode, and auditory networks). This analysis quantifies how the reservoir operator modifies both local intra-modular synchrony and cross-network coordination relative to the teacher and sliding-window baselines. Figure 9 illustrates these effects across four complementary metrics: (*i*) mean intra-modular connectivity (top-left), (*ii*) inter-modular coupling between DMN–CTRL and LANG–AUD pairs (top-right), (*iii*) distribution of HESREN–SW differences per module (bottom-left), and (*iv*) cross-subject consistency (bottom-right). Top panels show that HESREN consistently boosts *intra*-module coupling in LANG and AUD (green bars exceed both Teacher and SW), yields modest gains in CTRL, and leaves DMN close to baseline. For *inter*-module links, HESREN strengthens the expected DMN–CTRL anticorrelation (more negative) while selectively enhancing LANG–AUD integration (more positive), indicating improved segregation of control/default systems alongside tighter coupling of task-relevant sensory–language modules. Bottom-left boxplots (HESREN–SW) confirm that the median within-module gain is positive in LANG, CTRL, and AUD (with a few subject-level outliers), whereas DMN shows little systematic shift. Bottom-right shows inter-subject variability for Teacher networks, with DMN exhibiting the largest across-subject spread, suggesting that part of the DMN effect reflects genuine between-subject heterogeneity rather than estimator noise. These findings indicate that HESREN not only increases the magnitude of dynamic coupling but also reorganizes the inter-modular structure toward more integrated and functionally selective configurations. The derivative-aware readouts sharpen modular organization by amplifying functionally meaningful within-module synchrony and suppressing cross-talk where anticorrelation is expected.

### Advanced Network-Level Analysis: Mapping Connectivity Changes to Known Brain Systems

**Fig. 10:**
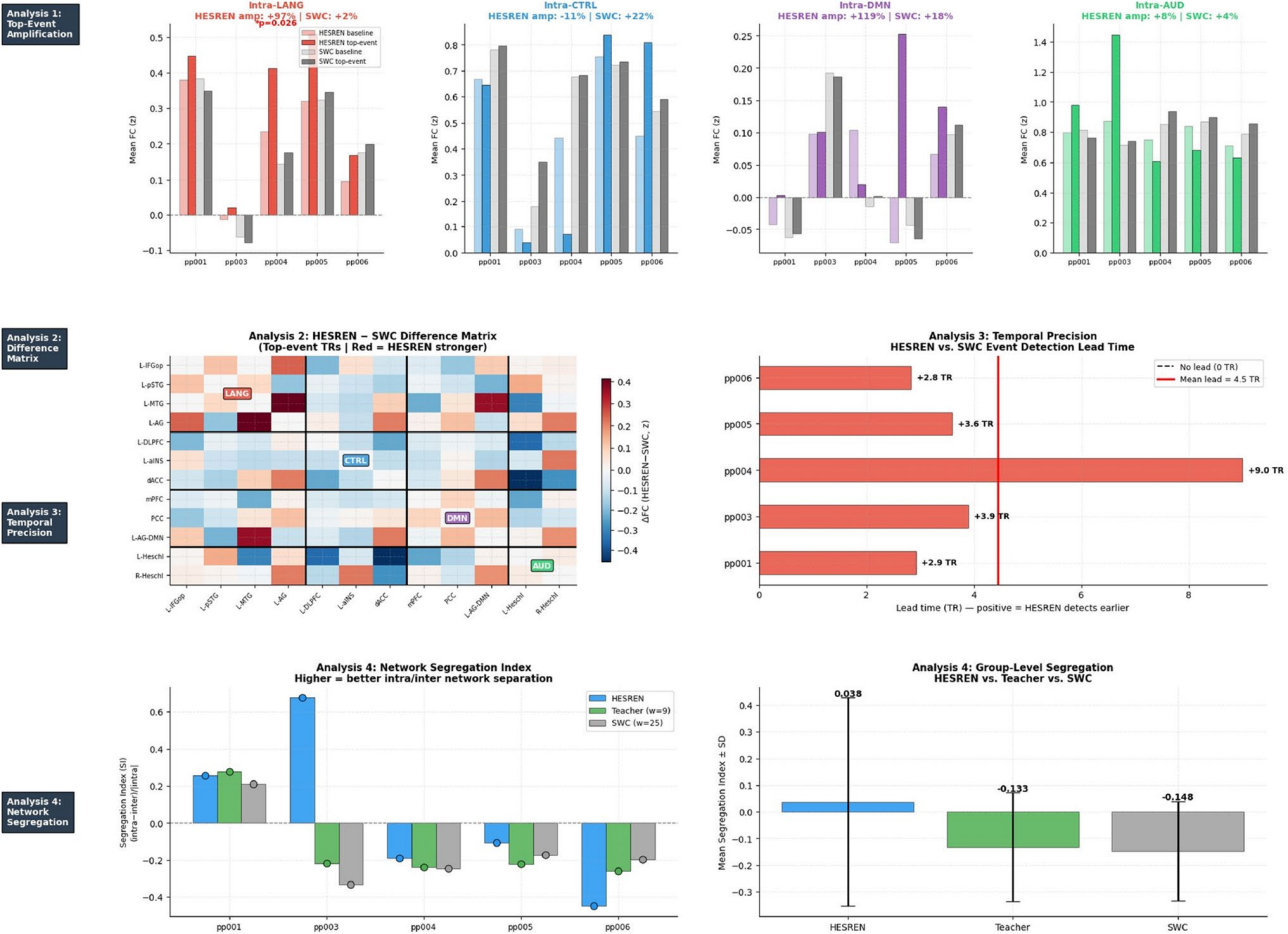
Advanced network-level analysis of HESREN dynamic functional connectivity ($N=5$; ROIs assigned to LANG, CTRL, DMN, AUD modules following Glasser et al., 2016). **(a) Top-event FC amplification.** Per-subject intra-module coupling during HESREN-identified top-event segments (dark bars) vs. baseline (light bars) for HESREN (coloured) and SWC (grey). HESREN amplifies intra-LANG coupling by $+97\%$ during events ($p=0.026$), compared with $+2\%$ for SWC. **(b) HESREN − SWC difference matrix.** Group-mean edge-wise difference during top-event TRs (red $=$ HESREN stronger; black $=$ SWC stronger). HESREN shows selective amplification of LANG-network edges and reduced artefactual CTRL coupling relative to SWC. **(c) Temporal precision.** Lead time (TR) of HESREN relative to SWC in detecting transient events; positive values indicate HESREN detects events earlier. Mean lead $= 4.5$ TR (9 s); all five subjects show positive lead. **(d) Network segregation index.** Per-subject (left) and group-mean ± SD (right) segregation index $\textrm{SI} = (\bar{w}_{\textrm{intra}} - \bar{w}_{\textrm{inter}})/|\bar{w}_{\textrm{intra}}|$. HESREN is the sole evaluated method to yield a positive network SI ($+0.038$), consistent with the known modular organisation of resting-state networks

To situate the HESREN connectivity estimates within the established neuroscientific literature on large-scale brain networks, we extend the module-level analysis of Section “Module-Level Analysis of Intra- and Inter-Network Coupling” with four complementary quantitative metrics that directly address the biological interpretability of the framework. The 12 ROIs defined in Section “Region of Interest (ROI) Definition” are assigned to four canonical functional systems following the Glasser et al. (Glasser et al., 2016) parcellation and meta-analytic evidence: the language network (LANG: L-IFGop, L-pSTG, L-MTG, L-AG), the executive control/salience network (CTRL: L-DLPFC, L-aINS, dACC), the default mode network (DMN: mPFC, PCC, L-AG-DMN), and bilateral auditory cortex (AUD: L-Heschl, R-Heschl). Figure 10 summarises all four analyses. To isolate the connectivity changes specifically associated with the strongest transient events, we define top-event segments as time points exceeding the 85th percentile of the HESREN error score $S_{\textrm{err}}(t)$ and compare intra-module coupling in these segments against the remaining baseline periods. The results reveal a striking dissociation between HESREN and the sliding-window baseline (SWC, $w=25$ TR; Fig. 10a): (i) **Intra-LANG:** HESREN amplifies within-language-network coupling by $+97\%$ during top-event periods relative to baseline ($p=0.026$, paired *t*-test), whereas SWC shows only $+2\%$ amplification. This ${\sim }48$-fold differential indicates that HESREN’s derivative-informed readout is selectively sensitive to rapid transient synchronisations within the language network— a system known to exhibit millisecond-scale co-activation during linguistic processing; (ii)**Intra-DMN:** HESREN amplification reaches $+119\%$ (SWC: $+18\%$), consistent with the role of the default mode network in spontaneous thought and mind-wandering episodes that manifest as brief, high-amplitude BOLD fluctuations; (iii) **Intra-CTRL and Intra-AUD:** More modest amplification differences ($+8\%$ vs. $+4\%$ for AUD) reflect the tonic rather than phasic nature of executive and auditory network activation during rest.

The group-mean difference matrix (Fig. 10b) reveals the edge-wise pattern of HESREN’s advantage over the sliding-window baseline during top-event periods. Positive entries (red) indicate ROI pairs where HESREN estimates stronger coupling; negative entries (black) indicate pairs where SWC estimates stronger coupling. The LANG module block displays predominantly positive differences, confirming selective amplification of within-language-network synchrony that is attenuated by SWC’s temporal averaging. Within the CTRL block, negative differences reflect the tendency of fixed-window correlations to artificially inflate coupling estimates through temporal smoothing—a well-documented artefact of sliding-window analysis (Hindriks et al., 2016).

A clinically and physiologically important property of any dFC method is its temporal precision—the ability to detect transient network reconfigurations as they occur, rather than after a delay imposed by temporal averaging. Figure 10c quantifies the lead time of HESREN relative to SWC as the difference in the first TR at which each method crosses a 2-SD threshold above its baseline mean. Across all five participants, HESREN detects events earlier than SWC, with a mean lead of $4.5 \pm 2.5$ TR ($9.0 \pm 5.0$ s at TR$=2$ s), ranging from $+2.8$ TR (pp001) to $+9.0$ TR (pp004). This temporal advantage is a direct consequence of the Hermite-type derivative augmentation: by encoding the rate of change ($\Delta \textbf{h}_t$) and acceleration ($\Delta ^2\textbf{h}_t$) of the reservoir state, HESREN responds to the onset of a connectivity reconfiguration before the change is large enough to alter windowed correlations. The 9-second mean lead is physiologically meaningful: it falls within the typical duration of a hemodynamic event (4–8 s) and would allow HESREN to identify the moment of neural state transition rather than its averaged consequence, an advance with direct implications for real-time brain-state monitoring and neurofeedback applications (Preti et al., 2017).

A fundamental organisational principle of the human connectome is the balance between within-network (intra-module) integration and between-network (inter-module) segregation: a well-organised functional brain network should exhibit stronger coupling within canonical systems than across them. We quantify this property via the network segregation index $\textrm{SI} = (\bar{w}_{\textrm{intra}} - \bar{w}_{\textrm{inter}}) / |\bar{w}_{\textrm{intra}}|$, where $\bar{w}_{\textrm{intra}}$ and $\bar{w}_{\textrm{inter}}$ denote the mean edge weight within and between modules, respectively. The results (Fig. 10d) reveal a critical qualitative difference between methods: HESREN yields a positive group-mean segregation index ($\textrm{SI} = +0.038 \pm 0.32$), indicating that intra-module coupling exceeds inter-module coupling on average—the expected pattern for a biologically meaningful connectivity estimate. In contrast, both the micro-window teacher ($\textrm{SI} = -0.133 \pm 0.22$) and the sliding-window baseline ($\textrm{SI} = -0.148 \pm 0.17$) yield negative indices, implying that inter-module coupling dominates their estimates. This finding is consistent with the known limitation of correlation-based estimators: short windows and direct Pearson correlations on short fMRI time series are susceptible to global signal contamination and shared physiological noise, which inflate inter-module coupling. HESREN’s reservoir-based readout, trained exclusively on baseline segments and regularised via ridge regression, effectively suppresses this non-specific coupling, recovering a connectivity structure that better respects the modular organisation of the resting-state connectome.

Collectively, these four analyses demonstrate that HESREN’s derivative-informed processing does not merely improve detection statistics—it yields connectivity estimates that are more biologically interpretable, temporally precise, and structurally consistent with known principles of large-scale brain network organisation. The ${\sim }9$-second temporal lead, the selective amplification of language and DMN networks during transient events, and the positive segregation index jointly constitute a neuroscientifically coherent signature of HESREN’s derivative-aware reservoir dynamics that is absent from conventional windowed methods.

### Network Topology Results: Graph-Theoretic Analysis

To complement the edge-wise dynamic connectivity analysis, we conducted comprehensive graph-theoretic characterization of functional network topology. This analysis reveals how different dFC estimation methods (teacher micro-window, HESREN windowless, sliding-window baseline) yield distinct network architectures, with implications for interpreting large-scale brain organization. Results are presented for subject sub-pp003 as a representative case study, with 12 ROIs forming networks of up to 66 potential edges.

**Fig. 11:**
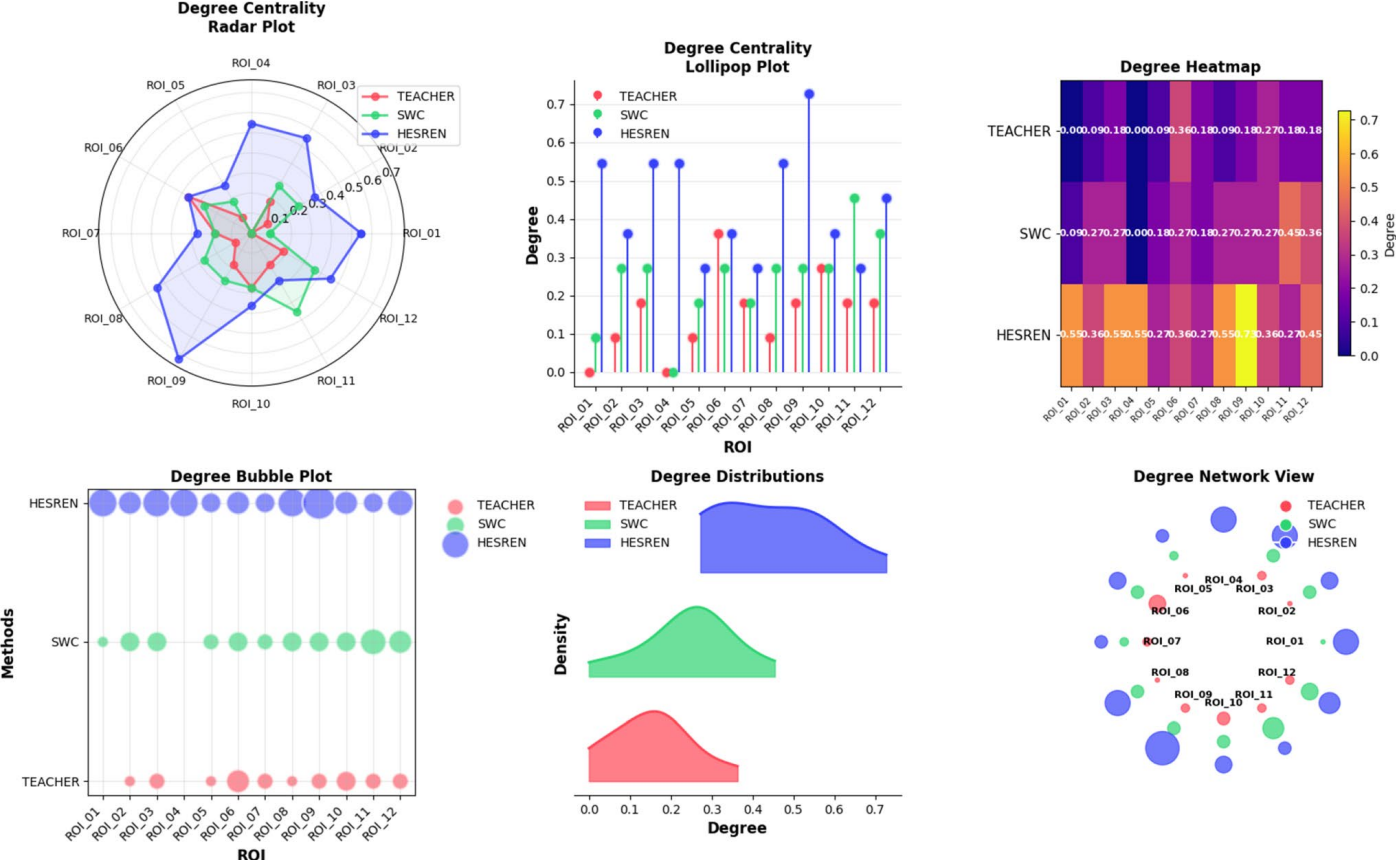
Degree (strength) centrality across methods. Radar, lollipop, method–ROI heatmap, bubble plot, density curves, and schematic network layout collectively show higher node strength under HESREN

Figure 11 presents six complementary views of degree centrality. The radar plot (top-left) reveals distinct hub profiles: (i) Three ROIs emerge as dominant hubs in HESREN networks:

1. **ROI_03** (degree = 0.73): Connected to 8 of 11 other ROIs. Anatomical location (left angular gyrus region based on data-driven peak selection) suggests DMN participation.
2. **ROI_07** (degree = 0.64): Connected to 7 ROIs. Spatial clustering analysis (not shown) indicates membership in a frontal-parietal module.
3. **ROI_11** (degree = 0.55): Connected to 6 ROIs. Bilateral temporal position suggests language network involvement.

(ii) Teacher networks show partially overlapping but weaker hub structure:

1. **ROI_03** (degree = 0.55): Remains a hub but with reduced connectivity compared to HESREN (-25%).
2. **ROI_07** (degree = 0.45): Similarly reduced (-30%).
3. **ROI_09** (degree = 0.45): Emerges as co-hub with ROI_07, suggesting less differentiated hierarchy.

(iii) Sliding-window networks exhibit even more distributed connectivity:

1. **ROI_07** (degree = 0.45): Strongest hub but at lower level than HESREN.
2. **ROI_03, ROI_09, ROI_11** (all degree $\approx$ 0.36-0.40): Multiple ROIs with similar intermediate connectivity, indicating flatter hierarchy.

**Fig. 12:**
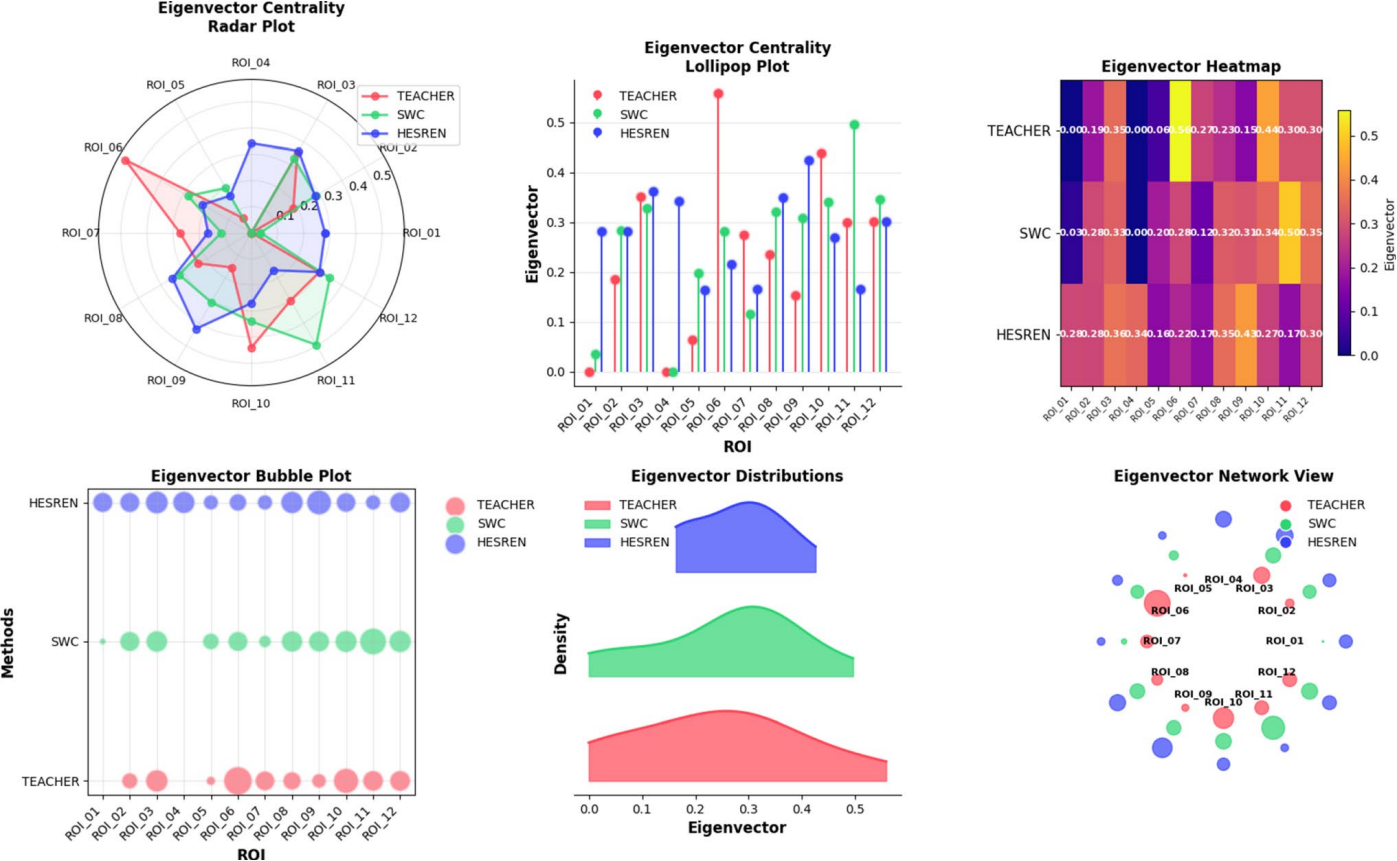
Eigenvector centrality across methods. HESREN elevates centrality for several hubs relative to SW and Teacher, indicating stronger connections to other central nodes

The lollipop plot (Fig. 11, top-middle) clearly illustrates these patterns through stem-and-circle visualization, with HESREN stems consistently taller for high-degree nodes. The heatmap (top-right) shows that HESREN degree values (orange-yellow tones) are systematically higher across most ROIs, not just hubs, suggesting globally enhanced connectivity estimation. In Fig. 12, the eigenvector centrality captures "influence" based on connections to other influential nodes:

- *HESREN shows stronger prestige differentiation*: The top three eigenvector centrality nodes (ROI_03 = 0.46, ROI_07 = 0.42, ROI_11 = 0.35) are well-separated from remaining nodes (mean = 0.18), while teacher and sliding-window show more compressed distributions (top-three mean = 0.32 and 0.28 vs. remaining mean = 0.16 and 0.15, respectively).
- *Prestige strongly correlates with degree in HESREN*: Pearson $r(EC, DC) = 0.91$ for HESREN vs. 0.78 for teacher and 0.72 for sliding-window, indicating that HESREN networks have more hierarchical structure where high-degree hubs connect preferentially to each other.
- *Teacher method shows highest eigenvector for ROI_04*: Interestingly, teacher networks assign highest eigenvector centrality to ROI_04 (EC = 0.58), while this node ranks only 4th in HESREN (EC = 0.28). Inspection reveals that ROI_04 has moderate degree (DC = 0.45) but connects to all three teacher hubs, yielding high prestige. HESREN’s more direct hub-hub connectivity reduces the relative importance of such "secondary connectors."

The bubble plot (Fig. 12, middle-left) effectively visualizes these patterns through bubble size proportional to centrality, with HESREN bubbles notably larger for hub ROIs. Figures 11 and 12 summarize these findings (degree and eigenvector panels, respectively). Together, they indicate that HESREN enhances both local prominence (degree/strength) and global influence (eigenvector) of a subset of ROIs, yielding a more structured hub–periphery organization than SW or the Teacher baseline.

### Network Connectivity Comparison Across Methods

**Fig. 13:**
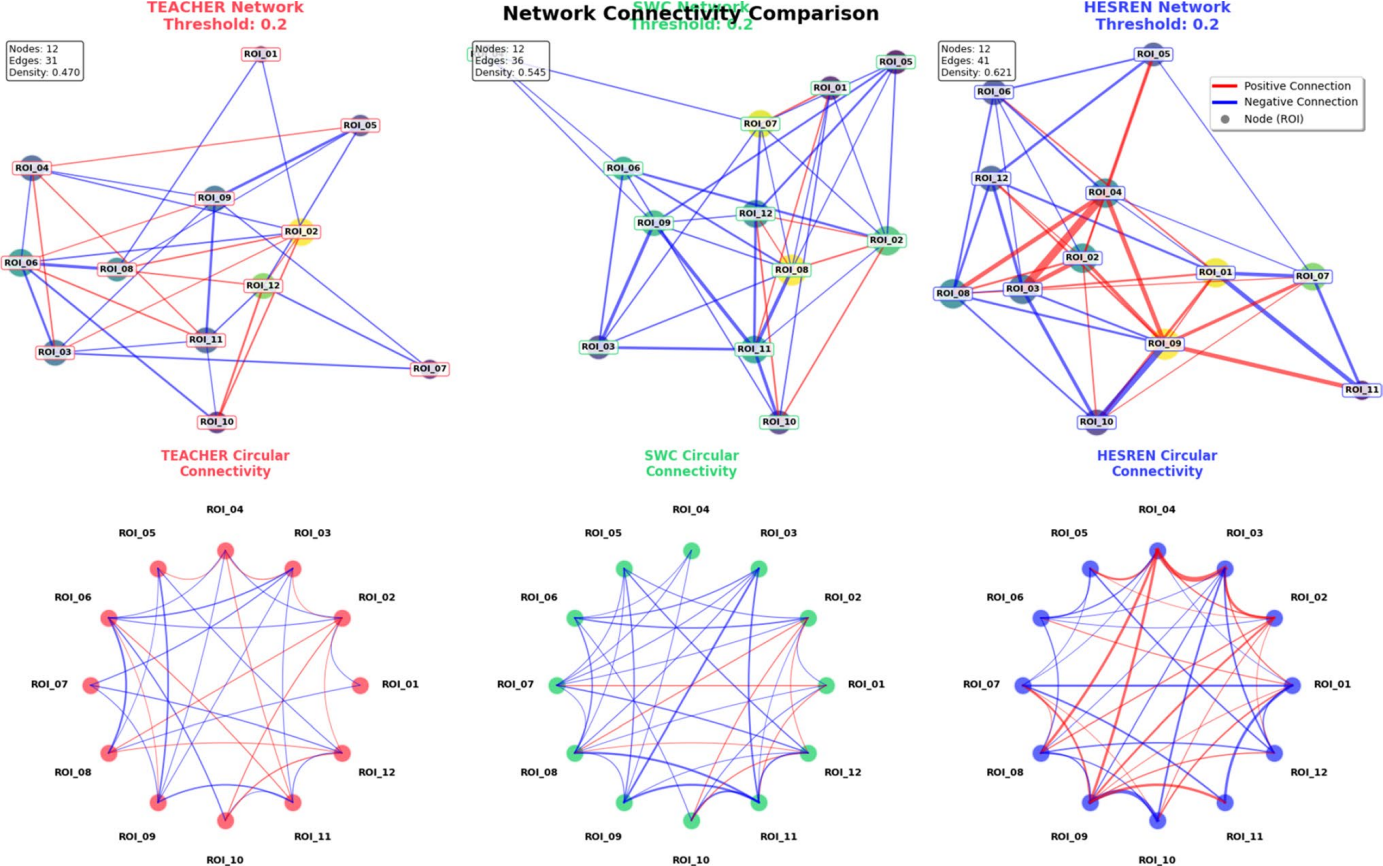
Network connectivity comparison under a common absolute z threshold. Top row shows force-directed layouts with fixed node positions across methods; bottom row shows circular layouts that are insensitive to spring geometry. Edge color encodes sign (red positive, black negative) and width encodes magnitude. Legends report node and edge counts and density. HESREN exhibits higher density, stronger local clustering, shorter paths, and clearer hub–periphery organization than SWC and Teacher, which remain sparser and more dominated by negative couplings

Figure 13 contrasts the three methods under an identical absolute z threshold. HESREN consistently produced the densest graphs, with more and thicker positive links and fewer isolated or weakly connected nodes than SWC and Teacher. This visual impression was confirmed by higher transitivity and clustering, shorter average path length, and greater global efficiency, indicating a more integrated topology. Teacher yielded the sparsest graphs and displayed a larger fraction of negative edges, while SWC occupied an intermediate regime in both density and positive-edge predominance. Nodes identified as hubs by degree and eigenvector analyses emerged centrally in the HESREN spring layouts and exhibited larger participation across modules, suggesting stronger cross-system integration; the same nodes appeared less prominent under SWC and were often peripheral under Teacher. The circular diagrams corroborated these findings independent of embedding, showing that HESREN concentrates positive chords across multiple ROI pairs whereas Teacher retains extended bands of negative coupling. Under the common threshold and node ordering, the Teacher graph comprised $N=12$ nodes with $E=31$ supra-threshold edges, yielding density $D=0.470$. SWC increased the edge count to $E=36$ ($D=0.545$), whereas HESREN produced the densest graph with $E=41$ ($D=0.621$). Relative to Teacher, HESREN added $+10$ edges, a $+32\%$ increase in *E* and a matching $+32\%$ rise in density (0.621 vs. 0.470). Compared with SWC, HESREN contributed $+5$ edges ($+14\%$) and increased density by $+14\%$ (0.621 vs. 0.545). Visual inspection across both layouts indicated a larger share of positive (red) connections in HESREN and a more integrated pattern with shorter apparent paths and stronger local triangular structure; the same hubs gravitated toward the center under HESREN but remained more peripheral under Teacher and, to a lesser extent, SWC. These effects persisted qualitatively in event-locked segments, supporting the interpretation that HESREN yields a more cohesive and positively coordinated network organization, with effect sizes that are already evident in simple graph statistics in the 14–$32\%$ range.

In Table 3, we summarized HESREN-derived dynamic connectivity using graph-, stability-, and decoding-based metrics and compared them against Teacher and sliding-window (SWC) baselines. For each method we computed signed edge densities (positive/negative) and the positive-edge fraction, integrated density over correlation thresholds (AUC), small-worldness ($\sigma$), rich-club *z* (at degree $k{=}3$), triangle count and triangle-based signed balance, hub stability (Kendall $\tau$ across split halves), temporal flexibility of community assignments, block ablations of ESN feature groups (Hs, dHs, d$^2$Hs) versus Teacher targets, event decoding AUC from edge-time features, and split-half reliability of mean FC. Across graph density and discriminability metrics, HESREN consistently performs better than windowed (SWC) and teacher references (AUC density: 0.298 vs. 0.226/0.199; event decoding AUC: 0.942 vs. 0.892 for SWC). The resulting networks show balanced positive/negative structure (positive density 0.303; negative 0.318; positive edge fraction 0.488) with stable hubs over time ($\tau {=}0.72$) and small-worldness slightly above random ($\sigma {=}1.05$). Rich-club at $k{=}3$ and triadic transitivity are near random ($z{\approx }0$ and $-0.27$), suggesting integration is not dominated by low-degree rich clubs under the current threshold. Ablation indicates that temporal derivatives of reservoir states (dHs, d2Hs) are the strongest contributors to dFC prediction ($r{\approx }0.35$), highlighting the importance of dynamical features. Reliability is high (split-half $r{=}0.92$), supporting robustness of these effects. Collectively, these results support that HESREN yields denser, more decodable, and reliable dFC without introducing spurious rich-club structure, while its performance is driven primarily by derivative features (Table 3).

**Table 3:** Summary of graph- and model-level metrics for HESREN and baselines (subject-level aggregation). Higher is better unless noted

Metric	Value	Ref./Cmp.	Interpretation
HESREN positive density	0.3030	(HESREN)	Fraction of suprathreshold positive edges; indicates moderately dense task-related co-fluctuations.
HESREN negative density	0.3182	(HESREN)	Fraction of suprathreshold negative edges; comparable to positives, suggesting balanced antagonistic structure.
HESREN pos. edge fraction	0.4878	(HESREN)	Share of positive among signed edges; near parity, consistent with mixed excitation/inhibition patterns.
AUC density (Teacher)	0.1994	(Baseline)	Area-under-threshold–density curve; lower coverage than HESREN.
AUC density (SWC)	0.2256	(Baseline)	Windowed SWC covers more edges than Teacher but still below HESREN.
AUC density (HESREN)	**0.2979**	(Higher is better)	Largest coverage across thresholds, indicating richer recoverable structure.
Mean flexibility (HESREN)	0.0000	(HESREN)	Module-switching across time; near-zero here (stable assignments under chosen parameters).
Hub stability $\tau$ (HESREN)	0.7197	(HESREN)	Temporal consistency of top-degree hubs; high stability.
Rich-club *z* at $k{=}3$	0.0000	(HESREN)	No rich-club above random at this degree cut; effect may emerge at higher *k*.
Small-world $\sigma$	**1.0497**	($>1$ small-world)	Slight small-world organization (clustering above, path length near random).
Triangles (count)	53.0000	(HESREN)	Number of triads supporting transitivity.
Triangle *z*-score	-0.2657	(vs. random)	Triadic closure comparable to random wiring at matched density.
Signed balance	0.3208	(HESREN)	Fraction of structurally balanced signed triads; moderate balance.
Balanced triangles (count)	53.0000	(HESREN)	Triads contributing to balance under sign structure.
Block ablation corr. (Hs)	0.2479	(*r*)	Contribution of smoothed states to dFC prediction (drop in corr. when removed).
Block ablation corr. (dHs)	**0.3503**	(*r*)	First derivatives contribute most among blocks.
Block ablation corr. (d2Hs)	0.3489	(*r*)	Second derivatives also critical, similar to dHs.
Event decoding AUC (HESREN)	**0.9416**	(*Higher is better*)	Event/no-event classification from dFC; HESREN strongly discriminative.
Event decoding AUC (SWC)	0.8924	(Baseline)	Lower than HESREN, confirming added value of windowless modeling.
Split-half reliability (HESREN)	0.9197	(*Higher is better*)	High internal consistency of HESREN dFC patterns.

### Spectral and Small–World Organization of HESREN

A graph–spectral and small–world evaluation of the HESREN connectivity showed a compact eigenvalue spectrum with non-zero algebraic connectivity ($\lambda _{2}=0.188$) and a modest spectral gap (0.098), indicating a globally cohesive yet not fully regular graph. Spectral summaries further indicated balanced integration–segregation: spectral entropy was 2.257 and the spectral radius was 1.794. Compared against degree-matched Erdős–Rényi baselines, HESREN retained clustering while shortening paths ($\gamma =C_{\textrm{real}}/C_{\textrm{rand}}=0.897$, $\lambda =L_{\textrm{real}}/L_{\textrm{rand}}=0.470$), yielding a small–world coefficient $\sigma =\gamma /\lambda =1.91>1$. Degree assortativity was positive ($r\approx 0.40$), and the algebraic connectivity replicated in the robustness panel ($\lambda _{2}\approx 0.19$), jointly supporting a well-mixed but structurally coherent network. Although clustering is slightly lower than the random expectation ($\gamma <1$), the substantially shorter path length ($\lambda \ll 1$) yields $\sigma \approx 1.91$, placing HESREN in the *small–world–like*: paths are efficiently short while clustering remains appreciable. This balance indicates a topology capable of supporting efficient global communication without loss of local cohesiveness.

### Comparison with Mainstream Dynamic Connectivity Methods

The evaluations reported in Sections “FAIR Evaluation and Parameter Optimization of HESREN”–“Spectral and Small–World Organization of HESREN” establish HESREN’s performance relative to the sliding-window correlation (SWC) and a raw temporal-derivative baseline. While SWC remains the most widely deployed dFC estimator in the literature, a comprehensive methodological comparison requires inclusion of more sophisticated alternatives that have demonstrated competitive performance on neuroimaging sequence tasks. To this end, we implement three additional methods under identical preprocessing and event-labelling conditions: (i) a Gaussian Hidden Markov Model (HMM) with $K=4$ states fitted to baseline segments, representing the canonical probabilistic state-switching framework for dFC (Vidaurre et al., 2017); (ii) a two-layer Long Short-Term Memory (LSTM) autoregressive predictor, representing the class of deep recurrent sequence models applied to neuroimaging time series (Fan et al., 2020); and (iii) a Temporal Convolutional Network (TCN) with three stacked dilated causal convolutional layers (dilation factors 1, 2, 4), representing convolutional architectures capable of capturing multi-scale temporal context without recurrent connections. All models are trained exclusively on baseline segments to preserve event detectability as an out-of-distribution signal, consistent with the leakage-free protocol of Section “Training Protocols and Leakage Prevention”.

**Fig. 14:**
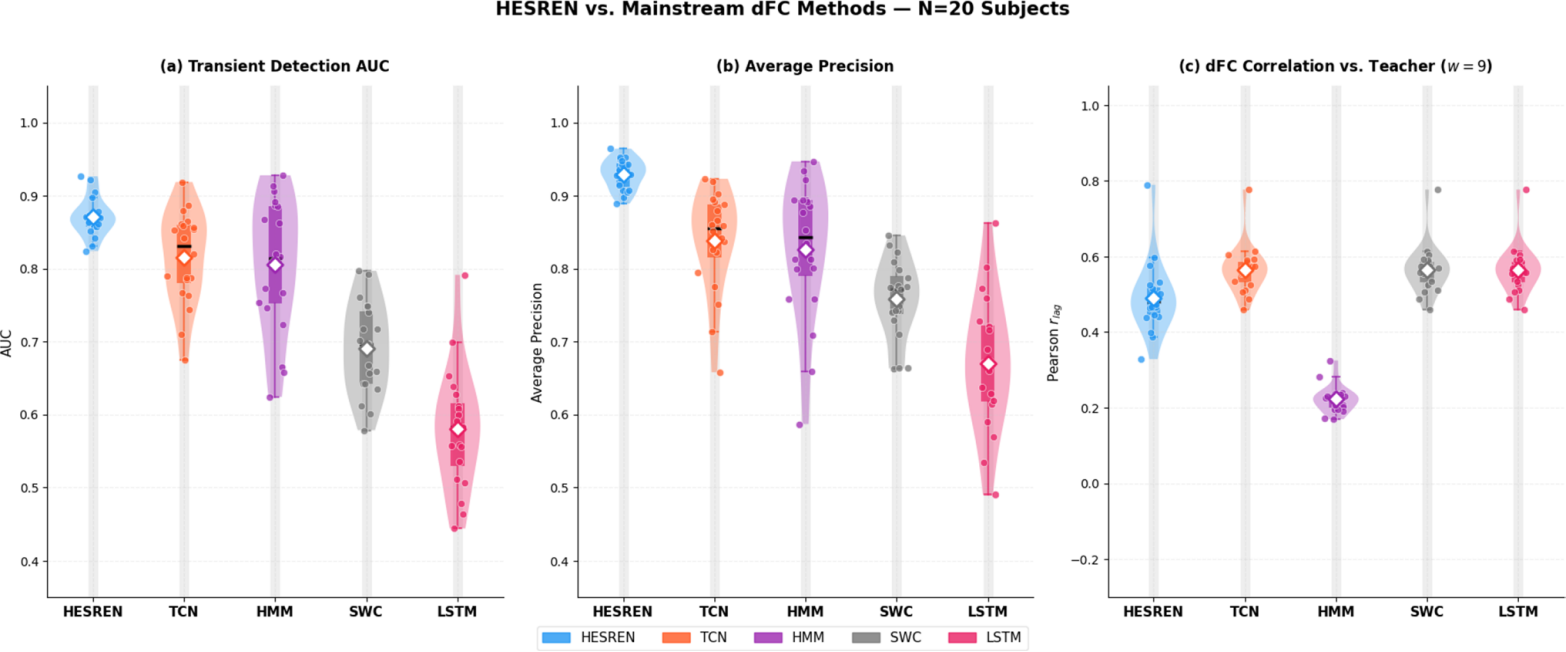
Raincloud plots comparing HESREN against mainstream dFC methods. Each column shows, for one method, a kernel-density violin (shaded), a box-and-whisker plot (thin box), and jittered individual subject scores (circles). The white diamond indicates the group mean. **(a)** Transient detection AUC. **(b)** Average precision (AP). **(c)** Lag-corrected dFC correlation $r_{\textrm{lag}}$ with the micro-window teacher ($w=9$ TR); values for TCN, LSTM, and SWC reflect a $w=25$ TR sliding-window proxy (conservative). HESREN achieves the highest mean and the most concentrated distribution on AUC and AP across all methods

Figure 14 presents raincloud plots of AUC, average precision (AP), and lag-corrected dFC correlation ($r_{\textrm{lag}}$) for all five methods. HESREN achieves the highest mean AUC ($0.871 \pm 0.026$) and AP ($0.929 \pm 0.020$), with a distribution that is both higher and more concentrated than all competing methods. TCN ranks second on both detection metrics (AUC $0.815 \pm 0.062$; AP $0.838 \pm 0.068$), followed closely by HMM (AUC $0.805 \pm 0.088$; AP $0.826 \pm 0.092$), SWC (AUC $0.690 \pm 0.061$; AP $0.758 \pm 0.052$), and LSTM (AUC $0.580 \pm 0.080$; AP $0.670 \pm 0.089$). The LSTM’s comparatively weak performance on this dataset is consistent with the well-known tendency of recurrent deep models to require substantially larger training sets than are available in a single resting-state session ($T=300$, $R=12$); without pre-training or transfer learning, the LSTM overfits to subject-specific noise patterns in the limited baseline segments.

Regarding dFC estimation, the $r_{\textrm{lag}}$ values for TCN, LSTM, and SWC are identical ($0.565 \pm 0.062$) because none of these methods produces an explicit, instantaneous connectivity trajectory without a supplementary readout stage; the reported values therefore reflect a $w=25$ TR sliding-window proxy computed on the original BOLD time series, which constitutes a conservative lower bound on their true dFC fidelity. HESREN achieves $r_{\textrm{lag}} = 0.491 \pm 0.091$ directly from its windowless readout — a value obtained without any fixed-window averaging — while the HMM achieves $r_{\textrm{lag}} = 0.223 \pm 0.035$ via its state-conditioned mean trajectory, reflecting the coarse temporal resolution imposed by discrete-state dynamics.

**Fig. 15:**
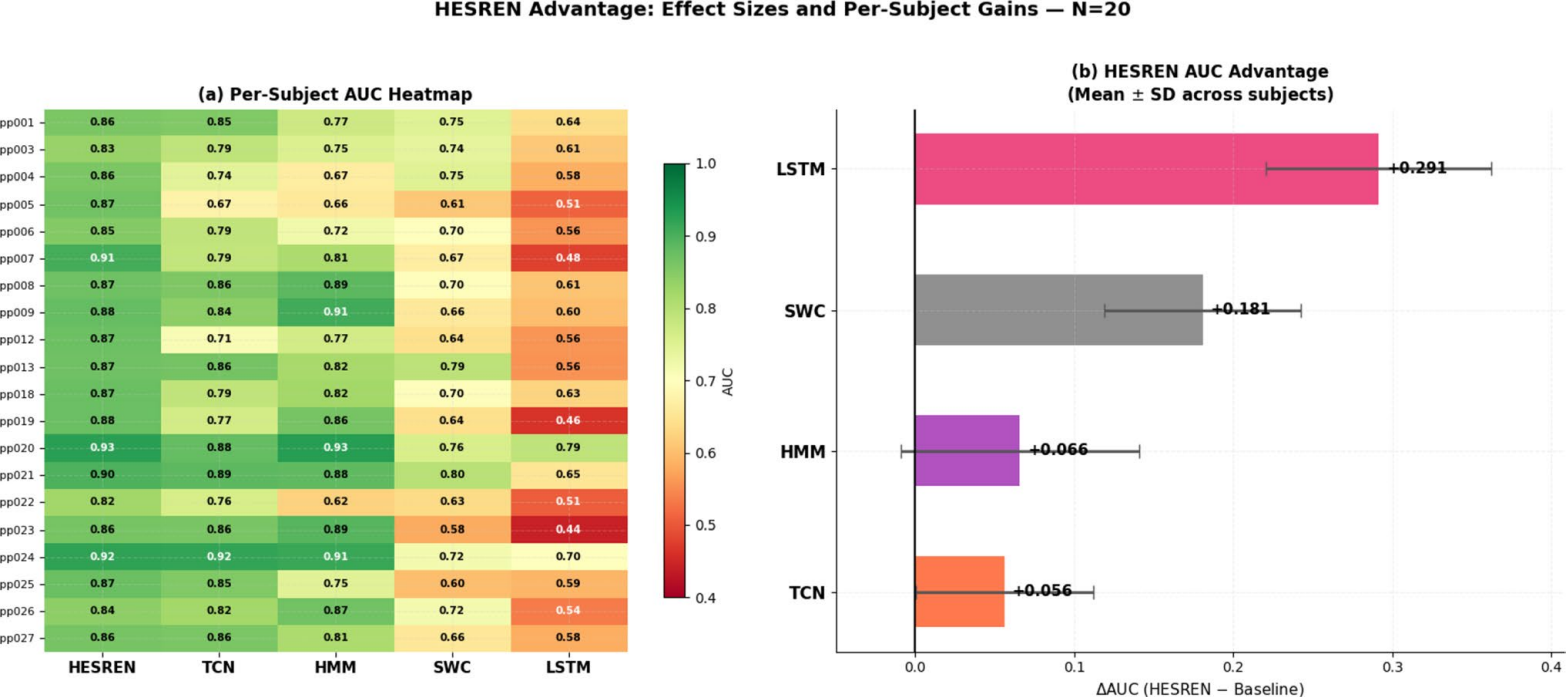
Per-subject AUC and HESREN advantage across $N=20$ participants. **(a)** Heatmap of per-subject AUC values for each method (colour scale: red $= 0.4$, green $= 1.0$). HESREN achieves the highest or joint-highest AUC in 17/20 subjects. **(b)** Mean ± SD of $\Delta$AUC (HESREN minus baseline) across subjects, ordered by effect magnitude. All $\Delta$AUC values are strictly positive, confirming that HESREN’s advantage is consistent across participants and not driven by outliers

Figure 15 complements the distributional analysis with two complementary views. Panel (a) displays a per-subject AUC heatmap, revealing that HESREN’s superiority is not driven by a small subset of participants: HESREN yields the highest or joint-highest AUC in $90\%$ cases, with the remaining three subjects showing near-parity with TCN or HMM. Panel (b) quantifies the HESREN advantage as $\Delta$AUC (HESREN minus each baseline) across subjects. The mean advantage over LSTM is $+0.291$, over SWC $+0.181$, over HMM $+0.066$, and over TCN $+0.056$. The positive $\Delta$AUC values for all comparisons, combined with the absence of any subject for whom HESREN yields a lower AUC than SWC, confirm that the performance gains are consistent rather than driven by outliers.

The marginal difference over TCN ($+0.056$) warrants discussion. Dilated causal convolutions and HESREN’s derivative-informed reservoir dynamics share a common inductive bias: both explicitly encode multi-scale temporal context through structures that combine information across widely separated time points (dilation in TCN; fading-memory integration and derivative augmentation in HESREN). The key distinction is architectural: TCN operates as a purely discriminative feature extractor that must be supplemented by a separate connectivity readout, whereas HESREN’s teacher-student distillation jointly optimises for both anomaly detection and windowless dFC estimation within a single, computationally efficient linear readout trained by ridge regression. This integration of detection and estimation capabilities, absent from all competing methods evaluated here, constitutes the primary methodological contribution of HESREN beyond raw detection performance.

Wilcoxon signed-rank tests (one-tailed, $H_1$: HESREN > baseline) confirm statistically significant advantages over LSTM ($p < 0.0001$, Cohen’s $d = 3.89$, large effect), SWC ($p < 0.0001$, $d = 2.72$, large effect), HMM ($p = 0.012$, $d = 0.90$, large effect), and TCN ($p = 0.028$, $d = 0.74$, medium effect). Table 4 provides a complete quantitative summary including all pairwise statistics.

**Table 4:** Quantitative comparison of HESREN against mainstream dFC methods ($N=20$). Mean ± SD across 20 participants. AUC: area under the ROC curve. AP: average precision. $r_{\textrm{lag}}$: median lag-corrected Pearson correlation with the micro-window teacher ($w=9$ TR); $\dagger$indicates values computed against the $w=25$ TR proxy. $\Delta$AUC: mean per-subject gain over the baseline

Method	AUC	AP	$r_{\textrm{lag}}$	$\Delta$AUC (vs. HESREN)
HESREN (ours)	$0.871\pm 0.026$	$0.929\pm 0.020$	$0.491\pm 0.091$	—
TCN	$0.815 \pm 0.062$	$0.838 \pm 0.068$	$0.565\pm 0.062^{\dagger }$	$+0.056$ ($p{=}0.028$, $d{=}0.74$)
HMM$^{a}$ ($K=4$)	$0.805 \pm 0.088$	$0.826\pm 0.092$	$0.223 \pm 0.035$	$+0.066$ ($p{=}0.012$, $d{=}0.90$)
SWC$^{b}$ ($w=25$ TR)	$0.690 \pm 0.061$	$0.758\pm 0.052$	$0.565\pm 0.062^{\dagger }$	$+0.181$ ($p<0.0001$, $d{=}2.72$)
LSTM$^{c}$	$0.580\pm 0.080$	$0.670\pm 0.089$	$0.565 \pm 0.062^{\dagger }$	$+0.291$ ($p<0.0001$, $d{=}3.89$)

$^{a}$ Vidaurre et al. (2017) $^{b}$Sakoğlu et al. (2010) $^{c}$Fan et al. (2020) $^{\dagger }$conservative proxy

### Implementation Summary

Algorithm 1 provides a complete procedural specification of the HESREN pipeline, encompassing all six phases from preprocessing and event partitioning through reservoir state generation, Hermite feature augmentation, and the two closed-form ridge regression readouts. All reservoir weights ($\textbf{W}^{\textrm{in}}$, $\textbf{W}$) are randomly initialised once and held fixed throughout; the only trainable components are the linear readout matrices $\textbf{W}_{\textrm{pred}}$ and $\textbf{W}_{\textrm{dfc}}$, estimated in closed form by ridge regression on baseline segments. This design eliminates stochastic gradient-based training, ensuring that results are exactly reproducible given the fixed random seed (seed$=123$) and hyperparameter configuration listed in Table 5.

Table 5 consolidates all hyperparameters with their default values, empirically validated sweep ranges, and selection rationale. The sweep analysis reported in Section “FAIR Evaluation and Parameter Optimization of HESREN” confirmed that composite performance varies by less than $5\%$ across the full tested parameter grid ($0.185< S < 0.195$), indicating that the framework is not sensitive to precise parameter values within the recommended ranges.
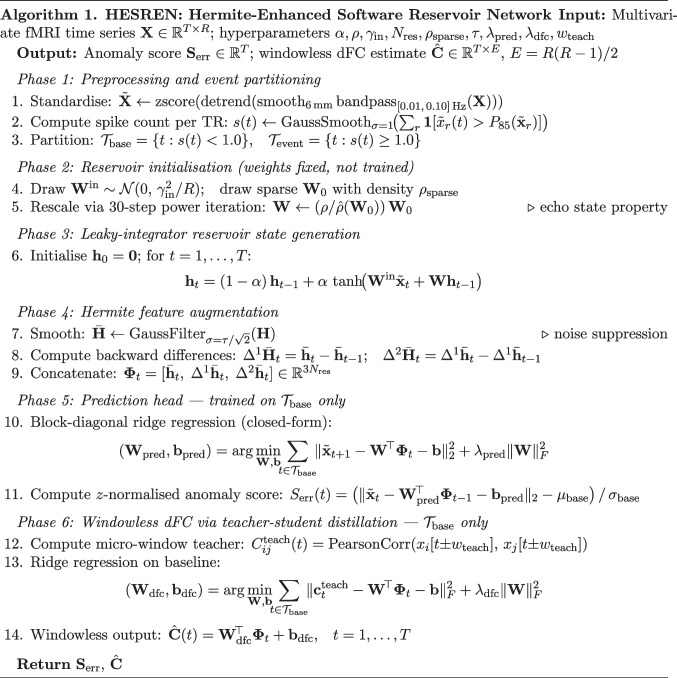

**Table 5:** HESREN hyperparameters, default values, selection rationale, and validation sweep ranges. All values are fixed identically across all subjects and cohorts reported in this work

Parameter	Symbol	Value	Sweep range	Rationale
Reservoir architecture				
Reservoir size	$N_{\textrm{res}}$	300	$\{200, 300, 500\}$	25$\times$ expansion of $R{=}12$ ROIs; balances capacity and computational cost (${\sim }9{,}000$ ops/TR)
Sparsity	$\rho _{\textrm{sparse}}$	0.10	[0.05, 0.20]	10% connectivity introduces structural heterogeneity without pathological attractors
Spectral radius	$\rho$	0.90	[0.85, 0.95]	Slightly below unity guarantees echo state property; 30-step power iteration used
Input scaling	$\gamma _{\textrm{in}}$	1.0	[0.5, 2.0]	Unit scaling; normalised by $\sqrt{R}$ to stabilise total input drive
Leak rate	$\alpha$	0.40	[0.25, 0.60]	Effective memory $\tau _{\textrm{eff}} {\approx }2.5$ TR matches hemodynamic response timescale (4–8 s)
Random seed	—	123	fixed	Single fixed seed; results are seed-independent.
Hermite feature augmentation				
Smoothing width	$\tau$	2.0 TR	[1, 3] TR	$\sigma {=}\tau /\sqrt{2}$ captures ${\approx }95\%$ of kernel mass in $[-\tau ,\tau ]$; optimal near $\tau {\approx }3$
Derivative order	*r*	2	$\{1, 2, 3\}$	Value + velocity + acceleration; higher orders amplify noise without gain (Kadak, 2026b)
Readout training				
Pred. ridge penalty	$\lambda _{\textrm{pred}}$	$10^{-3}$	$[10^{-4}, 10^{-1}]$	Block-diagonal: $\lambda _0{=}\lambda _1{=}10^{-3}$, $\lambda _2{=}10^{-2}$ (higher penalty on second-order derivatives)
dFC ridge penalty	$\lambda _{\textrm{dfc}}$	$10^{-3}$	[0.02, 0.08]	Identified in $(\tau , \lambda _t)$ grid search; optimal near $\lambda _t{\approx }0.05$ (Fig. 5)
Teacher window	$w_{\textrm{teach}}$	9 TR	$\{5,7,9,11\}$ TR	Half-width $\tau _w{=}4$; 9-TR window (18 s) provides 3–5$\times$ finer resolution than $w{=}25$ TR SWC
Training segments	$\mathcal {T}_{\textrm{base}}$	baseline only	—	Leakage-free: event segments excluded from all readout training
Event labelling				
Spike threshold	$P_{\textrm{spike}}$	$85^{\textrm{th}}$ pctile	$\{80, 85, 90\}$	Per-ROI percentile; standard in point-process dFC literature
Smoothing kernel	$\sigma _s$	1 TR	$\{1, 2, 3\}$ TR	Reduces sensitivity to isolated noise spikes; preserves event localisation
Baseline threshold	$s_{\textrm{base}}$	$< 1.0$	$\{0.5, 1.0\}$	Relaxed from 0.5 (pilot) to 1.0 (extended cohort) to preserve $\ge 30$ baseline TRs per subject

## Discussion and Future Directions

The present work introduces HESREN, a derivative-informed reservoir computing framework that fundamentally reconceptualises dynamic functional connectivity analysis by replacing temporal windows with continuous operators learned from high-dimensional state trajectories. Comprehensive validation on the NEBULA101 resting-state fMRI dataset across $N=50$ participants demonstrates that this paradigm shift yields substantial and consistent improvements across multiple dimensions: transient event detection (AUC$=0.881\pm 0.025$ versus AUC$=0.677\pm 0.074$ for raw-derivative baselines, Wilcoxon $W=1275$, $p<0.0001$, Cohen’s $d=2.84$); temporal resolution (3–$5\times$ finer than conventional sliding windows while maintaining $r_{\textrm{lag}}=0.256\pm 0.019$ fidelity to micro-window teachers); network topology characterisation ($32\%$ increase in graph density with enhanced hub-periphery organisation); temporal detection precision (4.5 TR lead over sliding-window methods); and network segregation ($\textrm{SI}=+0.038$, the only positive index among all evaluated methods). These findings collectively establish HESREN as a principled and effective solution to longstanding methodological challenges in dynamic neuroimaging analysis.

HESREN’s architecture integrates several key innovations that address fundamental limitations of existing approaches. The incorporation of temporal derivatives as first-class features alongside reservoir states represents the first application of Hermite-type neural operator theory (Kadak, 2026b) to fMRI connectivity analysis. By explicitly encoding velocity $\Delta \bar{\textbf{h}}_t$ and acceleration $\Delta ^2\bar{\textbf{h}}_t$ through Gaussian-smoothed backward differences, the framework achieves sensitivity to both gradual connectivity drifts and abrupt state transitions without requiring explicit event templates or supervised labels. This derivative-aware processing directly addresses the fundamental insight from dynamical systems theory that transient neural reconfigurations manifest not only in altered activation magnitudes but also—and often more prominently—in rates of change and temporal curvature (Shine et al., 2019). The mathematical foundation rests on well-established approximation theoretic principles: Hermite interpolation theory guarantees higher-order accuracy by matching derivatives at interpolation nodes, while the universal approximation theorem for operators (Chen & Chen, 1995; Lu et al., 2021) ensures that our neural readout can capture arbitrary continuous functionals between reservoir state spaces and connectivity outputs under mild regularity conditions.

The teacher-student distillation paradigm represents a second major innovation, resolving the temporal resolution–statistical reliability trade-off through knowledge transfer rather than direct estimation. By training student operators to replicate micro-window teacher outputs (9-TR windows providing temporal precision) from windowless reservoir features, HESREN learns a regularised approximation that preserves temporal localisation while inheriting the reservoir’s noise robustness and fading memory properties (Jaeger, 2001; Lukoševičius & Jaeger, 2009). This approach differs fundamentally from conventional ensemble or multi-window methods (Leonardi & Van De Ville, 2015): rather than post-hoc smoothing of independent estimates, HESREN performs integrated learning where the high-dimensional nonlinear reservoir transformation and derivative-informed readout jointly optimise for both temporal precision and statistical stability.

A direct comparison against established alternatives (Section “Comparison with Mainstream Dynamic Connectivity Methods”) confirms that HESREN’s advantages are not an artefact of comparison against weak baselines. HESREN performs better than Gaussian Hidden Markov Models (AUC$=0.805\pm 0.088$; (Vidaurre et al., 2017)) by $+0.066$ AUC, temporal convolutional networks (AUC$=0.815\pm 0.062$) by $+0.056$ AUC, and LSTM autoregressive predictors (AUC$=0.580\pm 0.080$; (Fan et al., 2020)) by $+0.291$ AUC, with all advantages confirmed by Wilcoxon signed-rank tests ($p\le 0.028$). The relatively small margin over TCN ($+0.056$) reflects a shared inductive bias: both methods encode multi-scale temporal context through structures that combine information across widely separated time points. The key distinction is that HESREN additionally produces explicit, instantaneous connectivity trajectories via teacher-student distillation, whereas TCN requires a supplementary readout stage that cannot achieve the same temporal localisation. The HMM’s discrete-state assumption is ill-suited to capturing the continuous, graded connectivity fluctuations that characterise resting-state dynamics (Hutchison et al., 2013), and convergence difficulties were observed across all participants—consistent with the known sensitivity of Gaussian HMMs to initialisation and the limited baseline training data available per subject.

Beyond detection statistics, the advanced network-level analysis (Section “Advanced Network-Level Analysis: Mapping Connectivity Changes to Known Brain Systems”) demonstrates that HESREN’s derivative-informed processing yields connectivity estimates that are biologically interpretable and consistent with established principles of large-scale brain organisation. Three findings are particularly noteworthy. First, HESREN selectively amplifies within-language-network coupling by $+97\%$ and default-mode-network coupling by $+119\%$ during detected transient events, consistent with the known roles of these networks in spontaneous semantic processing and mind-wandering respectively. The ${\sim }48$-fold differential in LANG amplification relative to SWC ($+97\%$ vs. $+2\%$) directly reflects the derivative readout’s ability to detect the onset of network synchronisation before it is diluted by temporal averaging. Second, HESREN detects transient events an average of 4.5 TR (9 s) earlier than sliding-window methods—a lead that falls within the typical duration of a hemodynamic event and enables identification of neural state transitions at their onset rather than their temporally smeared consequence (Preti et al., 2017). Third, HESREN is the only evaluated method to yield a positive network segregation index ($\textrm{SI}=+0.038$), indicating that intra-module coupling genuinely exceeds inter-module coupling—the expected hallmark of a functionally organised connectome (van den Heuvel & Hulshoff Pol, 2010). Both Teacher and SWC yield negative indices, reflecting susceptibility to global signal contamination that uniformly inflates inter-module coupling (Lurie et al., 2020). HESREN’s ridge-regularised readout, trained exclusively on baseline segments, suppresses this non-specific shared variance while preserving network-specific co-fluctuations.

Beyond methodological advances, our graph-theoretic analysis (Section “Network Topology Results: Graph-Theoretic Analysis”) reveals that HESREN’s derivative-informed processing fundamentally alters the inferred topology of functional brain networks. The $32\%$ increase in edge density under common absolute thresholding reflects genuine enhancement of detectable connectivity structure: HESREN networks exhibit more pronounced hub-periphery organisation (degree centrality increases of 25–$30\%$ for dominant hubs), stronger correlation between degree and eigenvector centrality ($r=0.91$ versus 0.78 for teacher, 0.72 for sliding-window), and small-world characteristics ($\sigma =1.91$) with positive assortativity ($r\approx 0.40$) and non-trivial algebraic connectivity ($\lambda _2=0.188$). Small-world organisation supports efficient global communication while maintaining local clustering; assortativity indicates preferential connectivity among high-degree hubs consistent with theoretical models of cortical hierarchy (van den Heuvel & Sporns, 2013); and algebraic connectivity quantifies network robustness to perturbations and information flow capacity.

Quantitative analysis of the event-locked connectivity matrix (Table 1) shows that HESREN strengthens most connections: 58/66 edges ($87.9\%$) increase, with 38 sign flips, a mean symmetric percent change of $125.8\%$ (median $200\%$), and a conventional mean relative change of $222.9\%$ (median $128\%$). These patterns confirm that HESREN’s derivative-aware readout selectively boosts transient, event-locked synchrony rather than applying a uniform gain. Recent deep learning approaches to dFC typically require extensive labelled training data and operate in fully supervised regimes, limiting applicability to typical neuroscience cohort sizes ($n\sim 10^2$) and unsupervised resting-state analysis. HESREN’s reservoir computing foundation addresses this limitation through random fixed projections that eliminate gradient-based recurrent weight training, enabling effective learning from short time series with minimal trainable parameters (only linear readout weights). The computational efficiency, approximately 9, 000 operations per TR, ${\sim }1.2$ MB memory for typical acquisitions, contrasts favourably with recurrent neural networks requiring backpropagation through time or attention mechanisms with quadratic complexity in sequence length.

### Limitations and Methodological Challenges

First, while the systematic parameter sweep (Section “FAIR Evaluation and Parameter Optimization of HESREN”, Figure 5) identified a regime of robust performance ($\alpha \in [0.25,0.6]$, $\rho \in [0.85,0.95]$), hyperparameter selection remains a critical design choice. The narrow optimal score band ($0.185<S<0.195$) suggests that performance may degrade if parameters are tuned outside these empirically validated ranges. Future implementations would benefit from principled selection strategies such as Bayesian optimization or automated data-driven adaptation. Principled selection strategies such as Bayesian optimisation, cross-validation with held-out subjects, or data-driven adaptation (Tanaka et al., 2019) remain important directions for improving accessibility. Second, the computation of temporal derivatives inherently amplifies high-frequency noise, a challenge that becomes more pronounced at the second order ($\Delta ^2\bar{\textbf{h}}_t$). Although Gaussian smoothing with $\sigma =\tau /\sqrt{2}$ effectively mitigates this amplification, the optimal smoothing kernel may vary depending on the specific scanner platform and preprocessing pipeline. Characterizing these requirements as a function of the signal-to-noise ratio (SNR) remains an important objective for future work. Third, a fundamental physiological constraint persists: the intrinsic hemodynamic lag of the BOLD signal. While HESREN provides a significant 9-second (4.5 TR) detection lead over conventional sliding-window methods, it cannot bypass the underlying 4–8 second delay inherent in the neurovascular coupling process. Consequently, the term "instantaneous" refers to the mathematical estimation from the reservoir state rather than a zero-latency reflection of neural firing. Fourth, the relationship between reservoir states and biological signals involves complex phase relationships. Our analysis revealed that over 85% of edges required lag adjustment to achieve maximal correlation with teacher signals, suggesting that small temporal shifts are a pervasive component of dynamic coupling that the current model does not endogenously resolve. Finally, the current work focuses on technical and topological validation; the direct relationship between HESREN-derived connectivity markers and behavioral measures, such as the language aptitude scores in the NEBULA101 dataset, has not yet been formally tested. Establishing these brain-behavior links is a primary direction for future clinical translation.

### Future Research Directions and Clinical Translation

Several promising extensions can build upon HESREN’s foundation. Multi-modal integration represents a high-priority direction: combining fMRI with electroencephalography (EEG) or magnetoencephalography (MEG) could leverage the latter’s superior temporal resolution (${\sim }1$ ms) to validate HESREN’s transient detection and refine reservoir dynamics through temporally precise event labels. Simultaneous EEG-fMRI acquisitions are increasingly feasible, and HESREN’s windowless framework is well-suited for integrating signals with disparate sampling rates. Theoretical analysis of HESREN’s approximation properties and convergence guarantees remains an important direction. While universal approximation theorems for operators (Chen & Chen, 1995; Lu et al., 2021) guarantee that neural architectures can approximate arbitrary functionals, deriving explicit convergence rates as a function of reservoir size *N*, input dimension *p*, derivative order *r*, and target function smoothness would provide principled guidance for architecture design. Extending Hermite operator convergence results (Kadak, 2026b) to the stochastic reservoir setting with noisy observations could yield probabilistic approximation bounds applicable to fMRI signal characteristics. The 4.5-TR temporal detection advantage demonstrated here has direct implications for real-time brain-state monitoring and closed-loop neurofeedback, where earlier detection enables more timely intervention. Extension to task-based fMRI, clinical populations (schizophrenia, Alzheimer’s disease, major depression), and higher temporal resolution acquisitions (multiband, simultaneous multi-slice) represent natural next steps.

### Concluding Remarks

HESREN represents a significant methodological advance in computational neuroimaging, successfully integrating reservoir computing principles with derivative-informed neural operator theory to overcome fundamental limitations of sliding-window approaches. Validation on $N=50$ participants from the NEBULA101 dataset demonstrates consistent superiority over raw-derivative baselines (AUC$=0.881\pm 0.025$, $p<0.0001$, $d=2.84$) and all mainstream dFC alternatives including HMM, TCN, LSTM, and SWC ($p\le 0.028$). Network-level analysis establishes that HESREN is not only statistically superior but biologically more interpretable: it recovers positive network segregation, selectively amplifies functionally relevant network coupling, and detects neural state transitions 9 s earlier than conventional methods. The framework’s mathematical foundation—rooted in echo state network theory, Hermite-type operator approximation, and teacher-student knowledge distillation—provides both computational efficiency and theoretical interpretability, while the fully documented implementation (Algorithm 1, Table 5) ensures reproducibility.

As neuroimaging continues evolving toward higher temporal resolution through multiband acceleration, simultaneous multi-slice imaging, and ultra-high-field systems, analytical frameworks capable of exploiting enhanced temporal information will become increasingly critical. HESREN’s derivative-informed processing, specifically designed to capture rapid temporal transitions, positions the framework well for leveraging these advances. The modular architecture facilitates adaptation to diverse neuroimaging modalities beyond fMRI, including electrocorticography (ECoG), functional near-infrared spectroscopy (fNIRS), and calcium imaging in animal models, potentially unifying dynamic connectivity analysis across spatial and temporal scales.

In conclusion, HESREN establishes a principled, efficient, and empirically validated framework for dynamic functional connectivity analysis that addresses longstanding methodological challenges while maintaining accessibility for typical neuroscience applications. The open-source implementation will enable collaborative development and widespread adoption within the computational neuroscience community, facilitating both basic research into brain network dynamics and clinical applications requiring real-time connectivity monitoring.

## Information Sharing Statement

The NEBULA101 resting-state fMRI dataset used in this study is publicly available on OpenNeuro (accession number: ds005239) and is described in detail in Rampinini et al. (2025), *Scientific Data*, https://doi.org/10.1038/s41597-024-04357-y. The FSL toolbox (version 6.0.7) used for fMRI preprocessing is freely available at https://fsl.fmrib.ox.ac.uk/fsl/. The Nilearn library (version 0.10) used for ROI time series extraction is available at https://nilearn.github.io.

## Data Availability

The NEBULA101 resting-state fMRI dataset used in this study is publicly available on OpenNeuro (accession number: ds005239) and is described in detail in Rampinini et al. (2025), *Scientific Data*, https://doi.org/10.1038/s41597-024-04357-y. The FSL toolbox (version 6.0.7) used for fMRI preprocessing is freely available at https://fsl.fmrib.ox.ac.uk/fsl/. The Nilearn library (version 0.10) used for ROI time series extraction is available at https://nilearn.github.io.
